# Allosteric Coupling between the Intracellular Coupling Helix 4 and Regulatory Sites of the First Nucleotide-binding Domain of CFTR

**DOI:** 10.1371/journal.pone.0074347

**Published:** 2013-09-18

**Authors:** Jennifer E. Dawson, Patrick J. Farber, Julie D. Forman-Kay

**Affiliations:** 1 Molecular Structure and Function Program, The Hospital for Sick Children, Toronto, Ontario, Canada; 2 Department of Biochemistry, University of Toronto, Toronto, Ontario, Canada; University of Saskatchewan, Canada

## Abstract

Cystic fibrosis is caused by mutations in CFTR (cystic fibrosis transmembrane conductance regulator), leading to folding and processing defects and to chloride channel gating misfunction. CFTR is regulated by ATP binding to its cytoplasmic nucleotide-binding domains, NBD1 and NBD2, and by phosphorylation of the NBD1 regulatory insert (RI) and the regulatory extension (RE)/R region. These regulatory effects are transmitted to the rest of the channel via NBD interactions with intracellular domain coupling helices (CL), particularly CL4. Using a sensitive method for detecting inter-residue correlations between chemical shift changes in NMR spectra, an allosteric network was revealed within NBD1, with a construct lacking RI. The CL4-binding site couples to the RI-deletion site and the C-terminal residues of NBD1 that precede the R region in full-length CFTR. Titration of CL4 peptide into NBD1 perturbs the conformational ensemble in these sites with similar titration patterns observed in F508del, the major CF-causing mutant, and in suppressor mutants F494N, V510D and Q637R NBD1, as well as in a CL4-NBD1 fusion construct. Reciprocally, the C-terminal mutation, Q637R, perturbs dynamics in these three sites. This allosteric network suggests a mechanism synthesizing diverse regulatory NBD1 interactions and provides biophysical evidence for the allosteric coupling required for CFTR function.

## Introduction

**Figure 1:**
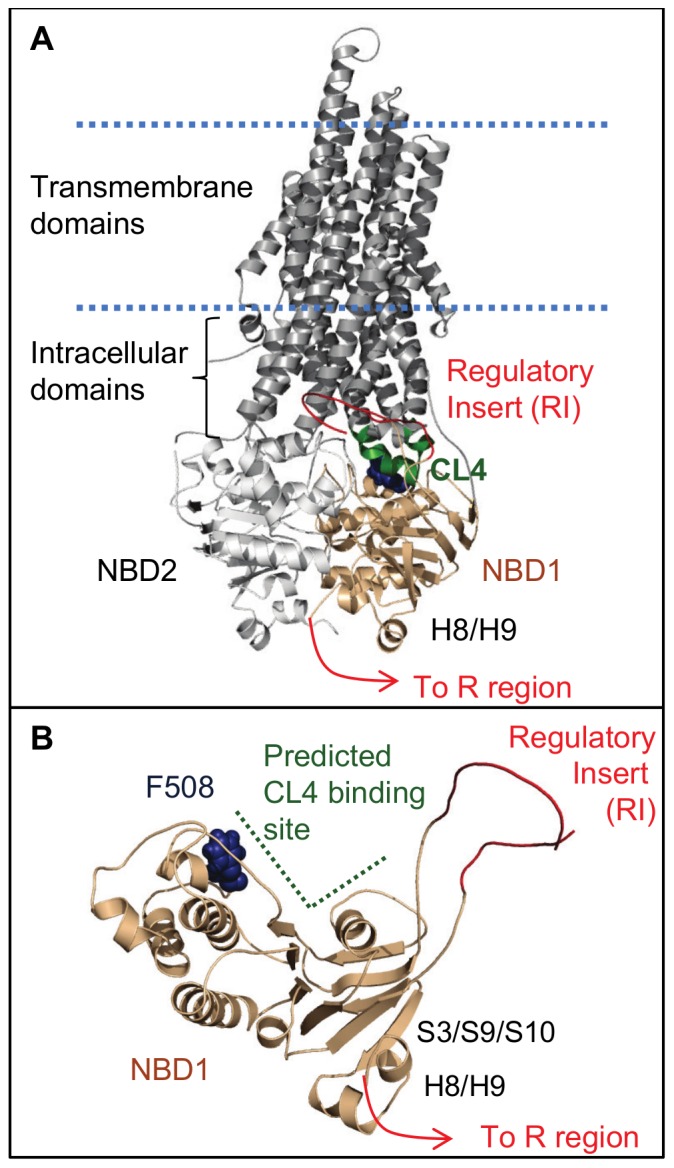
CFTR and its domains. A. Ribbon diagram of CFTR. NBD1 interacts with NBD2 and intracellular coupling helices, such as CL4. The disordered regulatory insert (RI) of NBD1 and the R region, which follows helices H8/H9 of NBD1, are sites of phosphor-regulation of the channel. B. NBD1 viewed from the NBD1:NBD2 interface. Residue F508 is in the CL4-binding site predicted by homology models [8], [9]. The figures were rendered with PyMOL [81] using a CFTR homology model [9]. The regulatory insert was modeled as a smoothed loop.

The CFTR (cystic fibrosis transmembrane conductance regulator) chloride channel is crucial to ion and fluid transport across epithelial cells [1]. Cystic fibrosis results from mutations in CFTR that compromise maturation or channel gating and can deleteriously affect many epithelia-lined organs, particularly the lungs, pancreas, and intestines. CFTR, a member of the ABC transporter family, contains two membrane-spanning domains, two intracellular domains composed of the extensions of the transmembrane helices into the cytoplasm, two cytoplasmic nucleotide-binding domains (NBD1 and NBD2), and a disordered regulatory (R) region that follows the C-terminal helices of NBD1 in sequence (Figure 1). The NBDs interact with four short helices in the intracellular domains, referred to as the coupling helices or loops (CLs), which are thought to couple changes of the NBDs to the rest of the channel. ATP binding and hydrolysis by NBD1 and NBD2 regulate channel gating [2]. PKA phosphorylation of the R region is a prerequisite for channel gating [3] and, in isolated domains, decreases R region interactions with NBD1 and NBD2 [4], [5]. The presence of mature CFTR on the plasma membrane depends on the correct intramolecular interactions, which guide the folding and processing of the channel. The CL4:NBD1 interface is especially important in the gating and maturation processes, with numerous CF-causing mutants located at this interface [6], [7]. The most common CF-causing mutation (CFTR1 database, www.genet.sickkids.on.ca), F508del, is in NBD1 within the CL4-binding site predicted by CFTR homology models [8]–[10]. This deletion disrupts CFTR folding on multiple levels, preventing maturation of the channel [11]–[13] by destabilizing NBD1 [14], [15] and disrupting the CL4:NBD1 interaction [16]. The maturation defects can be partially suppressed by mutations in NBD1 or the CL4 coupling helix, such as V510D and F494N/Q637R [11], [16]–[21], and by the drug VX-809 [22], [23], now in clinical trials. Removal of the disordered regulatory insert (RI) of NBD1 (Figure 1) both increases the stability of the domain [14], [24] and corrects the CL4:NBD1 interaction defect caused by F508del [16]. Many of the F508del-suppressor mutations, such as F494N, as well as the RI deletion, increase the solubility of the NBD1 domain [20], [24]. F508del lengthens the average time between CFTR channel openings [18], [25] and also attenuates the PKA-dependent activation of CFTR [26]. Both the solubilizing double mutant F494N/Q637R and the deletion of the RI increase the locked open time of WT and F508del CFTR binding pyrophosphate [27]. Pyrophosphate, in combination with ATP, is considered to prevent hydrolysis at the single efficient hydrolysis site of the NBDs, stabilizing the NBD1:NBD2 dimer and keeping the channel in its open conformation [28].

CFTR, like other members of the ABC transporter family, is allosterically regulated by nucleotide binding and interdomain interactions [2], [16], [29]. Binding of ATP or ATP-analogues between the NBDs changes the gating behavior and the populations of open and closed states observed in single channel studies [30]–[32]. Mutations to residues lining both sides of the CL4:NBD1 interface, a region distinct from the NBD1:NBD2 interface, also perturb channel gating [6], [7], suggestive of allostery. However, despite evidence that CFTR is allosterically regulated, there is little known about the allosteric networks themselves or even what regions of the domains they couple. Binding of ADP to the NBD of the homologous ABC transporter MJ1267 perturbs the dynamics at a remote site that corresponds to the CL4-binding site in CFTR, demonstrating allosteric coupling between the two regions [33]. Both CL4 and the ATP-binding sites are well-conserved in ABC transporters [1], [34].

CFTR has unique phospho-regulatory elements, the RI and the R region. In sequence, the R region follows helix H8 of NBD1 and may include its final helix, H9. A uniquely-conserved β-sheet (S3/S9/S10) in CFTR NBD1 interacts with H8 and H9, suggesting that this is an interaction site for at least part of the R region [35]. In addition to the expected allostery involving the ATP- and CL4-binding sites, we hypothesized allosteric communication between these CFTR-unique elements and other important regulatory sites within CFTR, such as the CL4:NBD1 interface. Ligand binding and mutations perturb the energy landscape of a protein [36]–[38]. If the CL4-binding site is allosterically coupled to the CFTR-unique elements, then the CL4:NBD1 interaction should perturb the phospho-regulatory elements and their local environment and, vice versa, phospho-regulatory changes should affect the CL4 interface. Mutations to any one of these regions may cause a reciprocal change to the other sites. Excess ATP was utilized in order to focus on CFTR-specific allostery by minimizing the contributions from the coupling of the ATP-binding site, in addition to stabilizing the NBD1 domain for NMR experiments.

With this as rationale, we probed effects of CL4 binding and mutations on NBD1 lacking the RI and identified a potentially CFTR-specific NBD1 allosteric network which links biologically relevant binding interfaces and regulatory regions. A mutation between C-terminal helices H8 and H9, Q637R, alters ^15^N-^1^H NMR HSQC spectral peak intensities that correspond to residues in the CL4-binding and RI-deletion sites, consistent with a change in dynamics within the spatially remote regions. Titration of a peptide containing the CL4 coupling helix into NBD1 leads to subtle changes of chemical shifts in the spectra. Combined probability correlation methods, in tandem with the precision of NMR chemical shift values, are profoundly sensitive to inter-residue correlations between changes in chemical shifts upon titration, even for relatively small chemical shift perturbations. These correlations reveal coupling within NBD1 between the predicted CL4-binding site, a C-terminal site containing β-strands S9/S10 and helices H8/H9 near the NBD1:NBD2 interface, and the RI-deletion site. Similar titration patterns were observed in NBD1 constructs containing Q637R and mutations near the CL4-binding site (F508del, F494N, and V510D), as well as in a CL4-NBD1 fusion. Altogether, results point to a dynamic allosteric network linking the NBD1:CL4 interaction to other regulatory and binding sites on NBD1, suggesting a possible mechanism for integrating NBD binding events to structural and functional consequences in the rest of the channel.

## Materials and Methods

### Reagent Constructs and Purification

The NBD1 ΔRI ΔRE constructs encode an isolated human NBD1 domain lacking the RI (CFTR residues 387–646, Δ405–436). For brevity, NBD1 ΔRI ΔRE shall be referred to as NBD1. All NBD1 constructs are in pET-SUMO vectors encoding kanamycin resistance and have an N-terminal His-SUMO purification tag. The CL4-NBD1 fusion construct {CL4(residues 1056–1076)-(SGGG)x5-NBD1 (residues 387–646, Δ405–436)} was designed by Structural GenomiX (SGX) and is currently available from the University of Arizona. Mutant NBD1 constructs with F494N, F508del, V510D, Q637R or F494N/Q637R were constructed with a Stratagene QuikChange site-directed mutagenesis kit. ^15^N-labeled CL4-NBD1 fusion was generously prepared in the lab of Christie Brouillette (University of Alabama at Birmingham). The other ^15^N-labeled protein samples were made using the previously published protocol [35]. The ^15^N-labeled 6His-SUMO used as a peptide binding control was obtained from Ulp1 cleavage of 6His-SUMO- wild-type (WT) NBD1.

The CL4 (residues 1057–1075), CL1 (residues 161–179), and CL3 (residues 958–968) peptides were synthesized by GenScript to >95% purity and used without further HPLC purification. Before use, the lyophilized powder was dissolved in ddH_2_O, pH adjusted to approximately 7 with NH_4_OH, and re-lyophilized to remove any trace amounts of organic solvents from purification.

Two buffers were used during NMR CL4 titration experiments, one optimized for NBD1 solubility and the other for CL4 solubility, both yielding similar CL4 titration results with elevated chemical shift changes in the predicted CL4-binding site and near the C-terminus of the protein (Figure S1). Both buffers contain 50 mM NaCl, 2% glycerol, 5 mM DTT, 5 mM MgCl_2_, 5 mM ATP, and 10% D_2_O; they differ in buffering compound and pH, 20 mM Tris-HCl, pH 7.5 or 20 mM sodium phosphate, pH 7.0. (The 1.0 mM F494N and F494N/Q637R NBD1 samples used 50 mM sodium phosphate to maintain consistent pH conditions.) For comparison, each set of CL-peptide:^15^N-labeled protein titration data set has a buffer, volume, and concentration-matched set of WT NBD1 titration spectra to ensure that their buffers were identical and the same amount of ligand was added to each protein. The protein concentration was 40 µM for all experiments, except in two cases. 1.0 mM F494N and F494N/Q637R NBD1 samples were used for peak intensity comparison. 65 µM CL4-NBD1 fusion was compared to a 1∶1 CL4 peptide:WT (isolated) NBD1 sample at the same concentration.

*NMR spectroscopy:* NMR experiments were performed at 20°C and the data were processed with nmrPipe [39].

#### Referencing

The chemical shifts are referenced to water. To confirm that the reference was not shifting during the titration, we found the RMSD for fifteen well-resolved and intense peaks that do not move during titration and were not used in the correlation analysis, mostly sidechain NH_2_ peaks. For the CL4:WT NBD1 data set, which contains five spectra, the ^1^H RMSD is 0.0019 ppm and the ^15^N RMSD is 0.016 ppm. As a comparison, residue 404, which has one of the smallest chemical shift changes during titration, moves 0.0030 ppm in the ^1^H dimension and 0.070 ppm in the ^15^N dimension, greater changes than due to potential referencing error.

#### Change of peak intensity due to mutation

TROSY ^15^N-^1^H HSQC [40], T_1_ [41], and T_1ρ_ [41] data were collected on 1 mM F494N, Q637R, and F494N/Q637R NBD1 samples using a cryoprobe-equipped 600 MHz Varian spectrometer. The chemical shifts and intensities of peaks in the HSQC spectra were determined using Sparky [42] and the T_1_ and T_1ρ_ relaxation data were fitted using FuDA [43] (available at http://pound.med.utoronto.ca/software.html). The T_1ρ_ and T_1_ data were then used to estimate the rotational correlation time of each protein [44] (using MATLAB R2006a for fitting).

#### Chemical shift changes due to ligand titration

Most of the ligand titration data were collected using ^15^N-^1^H HSQC experiments [45] on a cryoprobe-equipped 600 MHz Varian spectrometer. The exceptions are the second CL4:F508del NBD1 titration set and the CL1:WT NBD1 and CL3:WT NBD1 binding controls, which were recorded on an 800 MHz Varian spectrometer. The position of each residue’s peak in a 2D HSQC spectrum, (ω_1H_,ω_15N_), depends on the local chemical environment of that residue. The changes in chemical shift between NBD1 spectra are calculated by
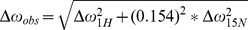
(1)in ppm, where Δω = ω(spectrum 2) − ω(spectrum 1) for ω_1H_ and ω_15N_. Spectra 1 and 2 are of WT and mutant NBD1 respectively for mutation data and are of apo NBD1 and CL4-bound NBD1 for titration data. The ^15^N chemical shifts are scaled, reflecting the ratio of the range of reported amide ^1^H and ^15^N chemical shifts from BioMagResBank [46]. The uncertainties in peak position for titrations of 40 µM WT NBD1 with the CL4 peptide were estimated by finding the standard deviation of each peak’s position,(ω_1H_, ω_15N_), in three repeated HSQC spectra of the same apo 40 µM WT NBD1 sample, then propagating the error by assuming that the uncertainties in apo peak position are roughly equivalent to those in the CL4-bound spectrum. This assumption would most likely result in an underestimation of the uncertainty because of the general decrease in peak intensities due to CL4 binding. The same process was repeated using three repeated HSQC spectra to find the uncertainties in peak position for apo 65 µM WT NBD1, 1∶1 CL4:WT NBD1 (65 µM peptide and 65 µM protein), and 65 µM CL4-NBD1.

## Results

### Q637R Mutation Alters Protein Dynamics in the RI-deletion Site and CL4-binding Site

In order to probe the molecular mechanism of allostery in CFTR NBD1, we monitored effects on NMR spectra of NBD1 ΔRI ΔRE (residues 387–646, Δ405–436) upon perturbation by mutagenesis and ligand binding. This construct, referred to as wild-type (WT) NBD1 for brevity, lacks the significantly disordered regulatory insertion (RI, residues 405–436) and the regulatory extension (RE), the first part of the regulatory (R) region. The lack of the RI leads to enhanced solubility and stability [24]. NBD1 binds ATP with K_d_ = 0.8 µM [24] and is both stabilized and solubilized by the presence of this nucleotide [14]. All experiments used 5 mM ATP to further maximize protein stability and to probe CFTR-specific allostery rather than consequences of nucleotide binding.

Observing the effects of perturbations, such as mutations or ligand binding, on NBD1 relies on the sensitivity of ^15^N-^1^H HSQC NMR spectra [45] to changes in the environment of the backbone amide groups. Each amide group has a peak whose position (chemical shift, ω) and intensity (I) are affected by neighboring groups, by the protein’s conformational ensemble, and by the dynamic motions of the protein. As previously observed [35], WT NBD1 has heterogeneous peak intensities across the spectrum. Weak, broadened peaks suggest µs-ms exchange between conformations. Other residues, like those near the RI-deletion site (residues 404, 437) and helices H8 and H9 (secondary structural elements numbered relative to NBD1 ΔRI ΔRE), are in fast exchange between conformations, leading to more intense, sharp peaks located at population-weighted average chemical shift positions. A heterogeneous ensemble, with exchange between conformations at multiple timescales, suggests that the free energy landscape of NBD1 is broad and rough [35]. Mutations and ligand binding can perturb the relative populations of the conformations, potentially affecting both local and remote regions via allosteric coupling [36]–[38]. Backbone resonances of WT and F508del NDB1 have been assigned to 89% [47] and 82% [35], respectively. WT NBD1 assignments were transferred to the NBD1 mutants investigated. Any ambiguous assignments, generally corresponding to residues near the mutation sites, were excluded from analysis.

**Figure 2:**
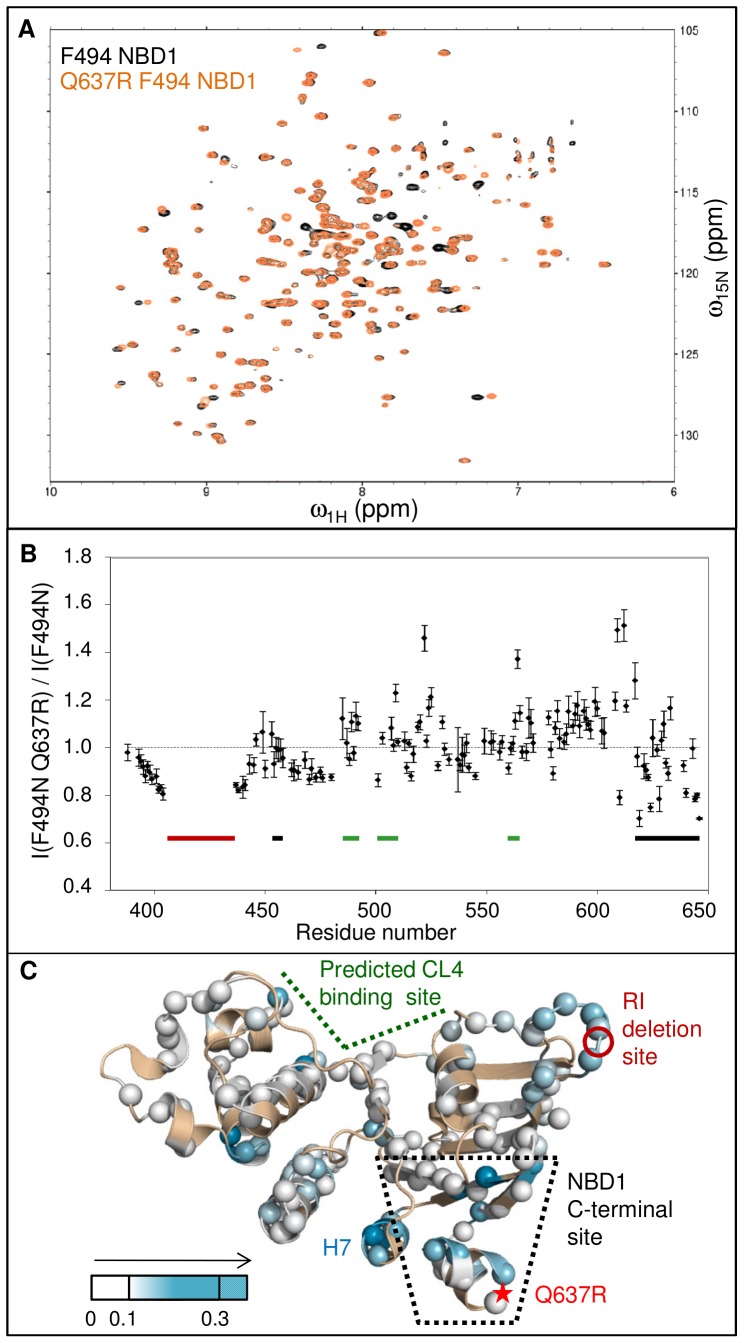
Change in peak intensity due to H8/H9 mutation Q637R. A. Overlay of F494N (black) and F494N/Q637R (orange) NBD1 HSQC spectra. B. Ratio of peak intensities for NBD1 containing the Q637R mutation to those of NBD1 without this mutation, reflective of changes in dynamics, as a function of residue. The approximate boundaries of the predicted CL4-binding site are indicated with a thick green bar and include β-strand S5, the C-terminal end of helix H2 and residues in the F508 loop (residues 485–492,501–510), and part of H5 (residues 560–565). The NBD1 C-terminal site (residues 453–458, 615–646) contains a β-sheet S3/S9/S10 and the C-terminal helices H8/H9 and is marked with a thick black line. The regulatory insert (RI, residues 405–436) is excluded from the NBD1 construct. A red line marks the deleted residues. Much of the Q loop (residues 492–502) remains unassigned, leading to missing information for these important residues at the NBD hetero-dimer interface. C. Dynamics changes (absolute value of the intensity ratio minus one) due to Q637R mapped onto the structure of NBD1, highlighting long-range effects in the RI-deletion site and CL4-binding site. Q637R effects were probed using F494N NBD1 for improved solubility and spectral quality. Figure rendered with PyMOL [81] using the NBD1 ΔRI ΔRE structure (PDB: 2PZE). View from the NBD1:NBD2 interface. (Unless indicated otherwise, all mapping figures use this structure.) The RI-deletion site is marked in red. Q637R, located between C-terminal helices H8 and H9, is marked with a red star due to lack of electron density in the structure.

Q637R is a solubilizing mutant that, in combination with F494N, partially corrects the folding defects induced by F508del in CFTR. Like WT NBD1, Q637R NBD1 has heterogeneous peak lineshapes (Figure 2A). To accurately quantify the change in peak intensity, concentrated (1 mM) NBD1 samples were required. A rotational correlation time of 16 ns was estimated for monomeric NBD1 by HydroNMR [48] based on the X-ray crystal structure, PDB ID 2PZE. At 1 mM, WT and Q637R NBD1 tumble in solution like objects that are larger than a monomer, with a rotational correlation time of τ_c_ = 27±5 ns (*A. Chong, unpublished data*) and 27±4 ns, respectively. The slower rotational times are consistent with self-association of the protein. The solubilizing mutation, F494N, reduces this association tendency. Both F494N NDB1 and F494N/Q637R NBD1 have a rotational correlation time of 22±2 ns. While still greater than the τ_c_ value expected for a monomer, the reduced correlation times result in improved spectral quality engendering greater confidence for determination of peak intensity ratios. The experimental equivalence of F494N and F494N/Q637R NBD1 τ_c_ values reduces the probability that any difference in lineshape may be caused by a difference in association states. Both decreases and increases in peak intensity, quantified by ratios of the intensities of the F494N/Q637R NBD1 and F494N NBD1 resonances (Figure 2B), are indicative of changes in dynamics. We mapped the absolute value of these changes (using Eq. 2) onto the NBD1 structure (Figure 2C).
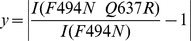
(2)


These changes in intensity point to the Q637R mutation affecting the dynamics of F494N NBD1 in multiple locations. The site of the Q637R mutations is located between the C-terminal helices H8 and H9 (residues 630–646), which are adjacent to a sheet containing β-strands S3/S9/S10 (residues 453–458, 615–629). These two helices and three strands are referred to here as the NBD1 C-terminal site. A number of residues near the mutation display altered peak intensities, as well as those in H7, which neighbors the NBD1 C-terminal site. Residues flanking the RI-deletion site (residues 404 and 437) show a general decrease in peak intensity. The CL4-binding site predicted in CFTR homology models [8], [9] includes a part of H5 (approximately residues 560–564), as well as portions of the α-subdomain, S5 (residues 488–491) and parts of H2 and the loop containing F508 (approximately residues 501–510) (Figure 1B). A few residues in this region, including G509, have modestly elevated intensities. Additional perturbed intensities are found near residue 520. NBD1 C-terminal site residues and those of neighboring helix H7 also have altered dynamics. While the Q637R mutation only changes the chemical shifts of neighboring residues (Figure S2), it perturbs the intensities of resonances in the C-terminal site nearby the mutation position, as well as for remote peaks near the site of the RI deletion and for some residues within the predicted CL4-binding site [8]–[10] (Figure 1A). In addition, there are intensity changes for residues outside these regulatory and interactions sites (Figure 2B) that may link these regions or connect to the other regulatory elements (Figure 2C), such as the ATP-binding site. These perturbed intensities are the result of changes in the inter-conversion rates between different NBD1 conformations, pointing to evidence for an allosteric coupling network between the NBD1 C-terminal site, the RI-deletion site, and the CL4-binding site.

### CL4 Binding Perturbs Chemical Shifts in the Predicted CL4-binding Site and C-terminal Region of WT NBD1

To assay binding to WT NBD1, a CL4 peptide containing CFTR residues 1057–1075 was designed. The boundaries are based on sequence conservation [34] and predicted hydrophilicity [49] in order to include the short coupling helix that is predicted to bind NBD1 in CFTR homology models [8], [9] and is at the center of a cluster of CF-causing mutants that result in channel gating or folding defects [6], [7]. The peptide begins to precipitate at 0.4–0.5 mM, so our given concentrations are the upper limit of the amount of ligand in solution. Binding assays were performed with a very low NBD1 concentration (by NMR standards) of 40 µM to maximize the molar ratio of peptide:protein (12.5∶1 was obtained) and minimize the effects of NBD1 self-association.

**Figure 3:**
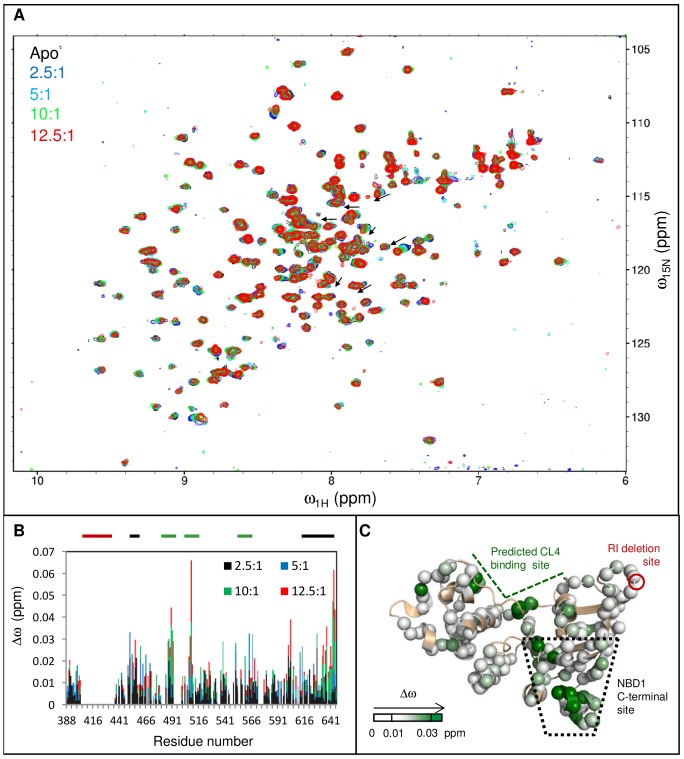
Chemical shift changes due to CL4 binding to WT NBD1. A. An overlay of the CL4:WT NBD1 titration spectra exhibits the relatively small chemical shift changes due to binding. A selection of well-resolved H9 residues are marked with arrows, indicating the shift toward random coil chemical shift values during titration. B. Δω_obs_ as a function of residue upon CL4 peptide (residues 1057–1075) binding for increasing concentrations of CL4 peptide, shows perturbations near F508 and the C-terminal helices H8 and H9. The CL4-binding site, the RI residues deleted from the construct and the NBD1 C-terminal site are indicated with thick bars above the chart that are colored green, red, and black, respectively. C. Δω_obs_ between spectra of apo and 12.5∶1 CL4 peptide:WT NBD1 are mapped onto the NBD1 structure using a white to green gradient (unassigned or unanalyzed residues in tan). The largest Δω_obs_ values are observed in the CL4-binding site predicted by homology models [8]–[10] and the NBD1 C-terminal site formed by strands S3/S9/S10 and helices H8/H9.

CL4 peptide binding to WT NBD1 perturbs the chemical shifts of a subset of residues (Figures 3A and 3B), indicating that binding occurs on the fast exchange timescale, altering the relative populations of the different conformations available to these residues. The chemical shift changes are relatively small; the greatest change is less than 0.07 ppm. Even at a molar ratio of 12.5∶1 CL4:NBD1, binding was not near to saturation with chemical shift changes linearly proportional to molar ratio (Figure S3). Due to the low signal-to-noise ratio and small shifts, changes for individual resonances are less accurate than the sum or average of all chemical shift changes. Some of the largest chemical shift changes, Δω_obs_, map to residues near F508 and to the cleft between the α-subdomain and the ATP-binding core (Figure 3C), consistent with the predicted CL4-binding site in CFTR homology models [8], [9]. Chemical shifts for residues in the S3/S9/S10 β-sheet and helices H8 and H9 of the NBD1 C-terminal site are also perturbed by CL4 binding. The resonances of helix H9 (residues 640–645) move downfield (to the left) during titration towards random-coil values of 8.5–8.0 ppm [50](Figure 3A, black arrows), suggesting a shift from helical conformations to a more disordered ensemble. Removal of H8/H9 would expose the S3/S9/S10 β-sheet, which is uniquely conserved in CFTR and has been implicated in binding of a CF modulator compound [35]. H9 may be part of the R region, which suggests that at least part of this phospho-regulatory region interacts with the unique β-sheet. The long-range effects observed here in the NBD1 C-terminal site due to CL4 binding near F508 support the presence of an allosteric coupling between the CL4-binding site and the CFTR-unique β-sheet beneath H8/H9. Together these data reinforce the intriguing suggestion of a link between the CL4-binding site and the very N-terminal end of the R region.

### CL4 binding Perturbs Chemical Shifts in the Predicted Binding Site and the NBD1 C-Terminal Site in Multiple NBD1 Constructs

The conclusion of allosteric coupling between the CL4-binding site and the NBD1 C-terminal site relies, in part, on relatively small perturbations of chemical shifts that are interpreted as long-range effects rather than direct binding to both sites. In order to test this conclusion, we monitored chemical shift changes upon CL4 binding to multiple different NBD1 constructs, as well as probed a CL4-NBD1 fusion protein for which direct binding of the CL4 to the C-terminal site is sterically hindered.

**Figure 4:**
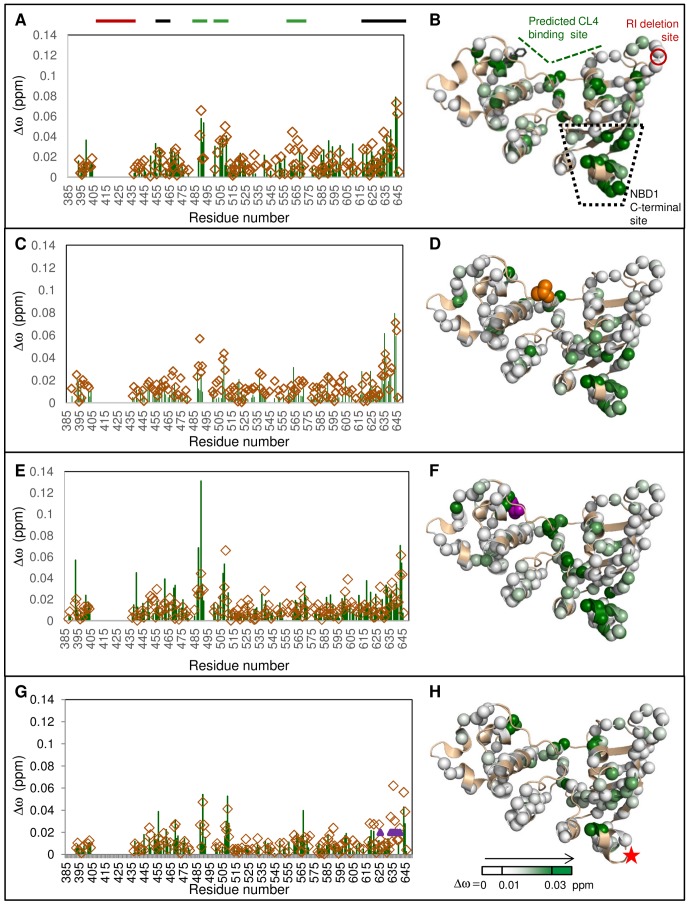
CL4 binding leads to similar chemical shift changes for NBD1 mutants. A, C, E, G. Δω_obs_ between spectra of apo and CL4 peptide:NBD1 are shown in green for F508del, F494N, V510D, and Q637R NBD1, respectively. The Δω_obs_ values for the buffer-matched CL4:WT NBD1 control spectra for each mutant are shown as open brown diamonds. Purple triangles in chart G indicate residues that could not be assigned for Q637R NBD1. The CL4:NBD1 proportions are 10∶1 for Q637R NBD1 and its CL4:WT NBD1 control, but is 12.5∶1 for the rest of the data. The thick bars above the charts indicate the CL4-binding site (green), the RI residues deleted from the construct (red) and the NBD1 C-terminal site (black). Δω_obs_ values are mapped onto NBD1 structure as in Figure 2: B B. F508del, D. F494N, F. V510D, and H. Q637R. F508del (position of F508 indicated as black sticks), F494N (orange space-filling representation), and V510D (purple space-filling representation) are near the predicted CL4-binding site. Q637R (red star) is between C-terminal helices H8 and H9.

The CL4 peptide binds to F508del NBD1, eliciting a similar pattern of chemical shift changes as observed for WT NBD1, with the largest Δω_obs_ centered on the predicted CL4-binding and NBD1 C-terminal sites (Figures 4A and 4B). CL4 also binds to NBD1 proteins containing single F508del-suppressor mutations, V510D and F494N, located near the predicted CL4-binding site, and Q637R, which lies between H8 and H9 (Figures 4 and S4). As expected for mutations in allosterically-coupled regions, they cause some changes to the overall pattern of residues perturbed by CL4 binding. CL4:F494N NBD1 binding causes smaller chemical shift changes in S5 (residues ∼488–491) than are observed in its buffer-matched CL4:WT NBD1 control spectra (Figures 4C and 4D). In contrast, V510D NBD1 has greater chemical shift changes in the CL4-binding site upon CL4 titration than WT NBD1, relative to changes in other residues (Figures 4E and 4F). Other differences are due to the lack of assignment for some residues near the site of the Q637R mutation (Figures 4G and 4H) and due to low spectral signal-to-noise. The low signal-to-noise and low CL4 solubility prevent quantitative comparison of the effects of mutation on CL4 binding. The overall CL4 binding pattern, with the greatest chemical shift changes located near the CL4-binding site and the NBD1 C-terminal site, remain consistent for all four mutants and for WT NBD1, suggesting the existence of an allosteric network between these sites that is not readily disrupted. Again, there are (generally smaller) chemical shift perturbations for residues outside these CL4-binding and C-terminal sites that may link these regions or connect to the other regulatory elements.

**Figure 5:**
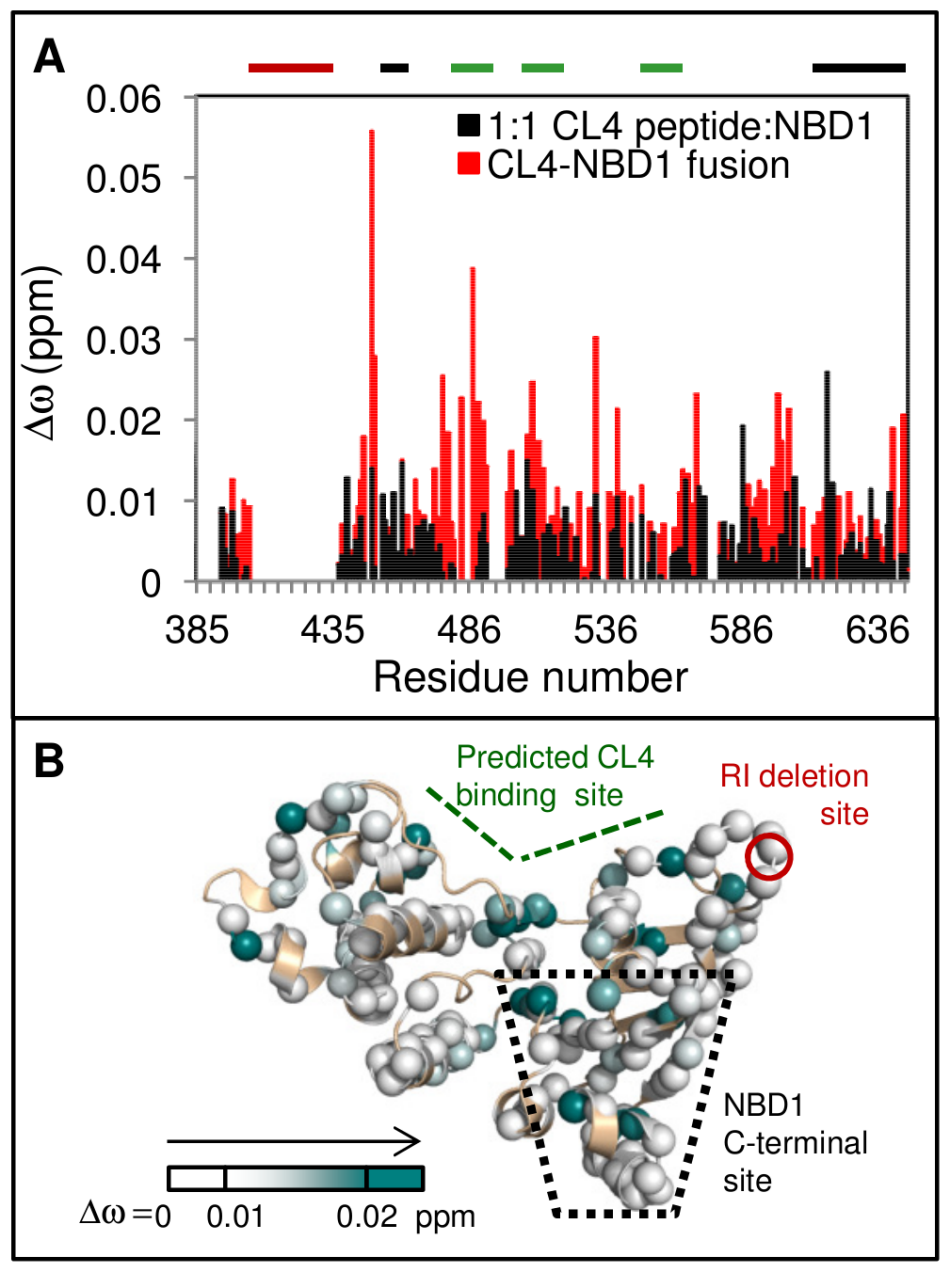
CL4 binding leads to similar chemical shift changes for a CL4-NBD1 fusion protein. A. Chemical shift differences, Δω_obs_, between the CL4-NBD1 fusion protein and NBD1(red) overlaid with Δω_obs_ for 1∶1 CL4 peptide:NBD1 (open black bars). There is an increase in Δω_obs_ in the CL4-binding and NBD1 C-terminal sites for the fusion protein, compared to the assay of the isolated reagents. The CL4-binding site, the RI residues deleted from the construct and the NBD1 C-terminal site are indicated with thick bars above the chart that are colored green, red, and black, respectively. B. CL4-NBD1 Δω_obs_ mapped onto NBD1 structure**.** A shallower white-to-teal gradient was used to map the Δω_obs_ values than was used, reflecting the smaller chemical shifts changes observed for intra-molecular CL4 binding within the fusion protein as compared to those for isolated NBD1 in the presence of a 12.5∶1 excess of CL4 peptide.

To address the possibility of direct CL4 peptide binding at both the CL4-binding and NBD1 C-terminal sites, we used a fusion protein, CL4-NBD1, containing a CL4 segment (residues 1056–1076) connected to WT NBD1 via a 20-residue (SGGG)×5 linker. The linker attaches to the N-terminus of NBD1, which is on the opposite side of the protein to the NBD1:NBD2 interface, making the 20-residue chain too short for CL4 to reach the C-terminal site, yet the intra-molecular binding pattern shows elevated Δω_obs_ in the CL4-binding site and NBD1 C-terminal site (Figure 5A), similar to the pattern observed using isolated NBD1 and CL4 polypeptides. These chemical shifts changes observed for intra-molecular CL4 binding within the fusion protein are twice as large as those for isolated NBD1 in the presence of a 1∶1 ratio of CL4 peptide (Figures 5A and S3), pointing to an effective concentration increase due to covalent linkage. There are additional chemical shift changes observed on the back of NBD1, most likely due to binding of the linker, which would tether CL4 closer to the predicted CL4-binding site. Thus, allosteric coupling is the most plausible explanation for the binding data. This allosteric coupling is strikingly robust, observed in five variants of isolated NBD1, in a CL4-NBD1 fusion protein, and even for CL4 segments that vary slightly in boundaries (residues 1057–1075 for the peptide versus 1056–1076 in the fusion protein).

When considering the same constructs, the binding pattern is also specific and repeatable across multiple data sets in different buffer conditions, even though CL4 has low solubility in both buffers and its binding to NBD1 is weak. Elevated chemical shift changes for residues in the CL4-binding site and the NBD1 C-terminal site are present in CL4:WT NBD1 titration data for samples in Tris buffer at pH 7.5 and those in sodium phosphate buffer at pH 7.0 (Figure S1). CL4:WT NBD1 samples in either buffer show significant chemical shift changes between residue 488–492 and from 501–510, which correspond to residues near or in S5, H2 or the F508 loop as predicted by homology models [8]–[10]. Only in sodium phosphate buffer are significant chemical shift changes observed for residues ∼560–564, which belong to the parts of H5 that are also predicted to belong to the CL4-binding site. Despite this difference and variations in the magnitudes of some Δω values in different buffers, the general features of the data are similar with elevated chemical shift changes within the predicted CL4-binding site and the NBD1 C-terminal site. The CL4 peptide does not bind SUMO, based on the lack of significant chemical shift changes upon titration (Figures S5 and S6). The CL1 peptide binding to WT NBD1 causes some larger (0.02 ppm or greater) chemical shift changes that are not clearly distinguishable from the noise (Figure S5B). While CL3:WT NBD1 binding does not result in significant chemical shift changes, there is possible broadening of many lower intensity peaks, which may be due to very weak binding of the peptide (Figure S6A). The perturbations of WT NBD1 upon CL1 or CL3 peptide binding were minor compared to the changes caused by binding of the CL4 peptide. As the CL3 and CL1 peptides were more soluble than the CL4 peptide in the buffer at the tested concentrations, the lack of binding cannot be attributed to poor solubility. Note that, for CL4, solid precipitate forms at concentrations greater than ∼0.4 mM, while CL1 was soluble at >0.5 mM for ∼4 hours at room temperature and CL3 was soluble at 0.8 mM for longer periods at the same temperature. Thus, CL4 binding to NBD1, while weak, appears to be specific with significant chemical shift changes observed at the direct CL4-binding site and allosteric NBD1 C-terminal site.

### Correlation Analysis of Titration Data Improves the Detection of Weak but Significant Changes due to CL4 Binding

CL4 binding causes relatively small Δω_obs_, the greatest of which is less than 0.14 ppm (Figure 4E; binding to V510D NBD1). The true concentration of CL4 in solution cannot be accurately determined near the peptide’s solubility limit and CL4:NBD1 binding is not saturated at this point. These issues complicate any analysis of the CL4:NBD1 interaction. The concentration and solubility limitations of the reagents eliminate the possibility of using ITC or fluorescence spectroscopy to characterize the binding or using an isotope-filtered NOE to directly identify the binding site. Efforts to conjugate a nitroxide label onto CL4 for paramagnetic relaxation enhancement experiments further decreased the solubility of the peptide (*data not shown*). The heterogeneity of NBD1 dynamics is a further confounding factor, since low-intensity broadened peaks near the noise level have increased experimental uncertainty for peak position. To overcome these challenges, a modified version of CHEmical Shift Correlation Analysis (CHESCA) [51] incorporating the Fischer combined probability test [52] was used, allowing small but significant peak shifts to be distinguished from random error (Figure S8, File S1).

Peak position, ω, can be affected by both proximal ligand binding and long-distance conformational changes that alter the relative populations within an ensemble. For a residue that is affected by ligand binding on the fast chemical exchange timescale, its peak position can shift in two dimensions during titration. If there is allosteric coupling between disparate regions of the protein, then chemical shift changes during titration should be correlated between affected residues. The real power of this analysis method lies in the detection of small, but significant, titrations that are not obvious from the chemical shift difference data (Figure S7 and S8). In this paper, an inter-residue correlation is considered significant if it has a one-tailed p-value less than or equal to 0.025. Since the criterion for significance is not zero, there is the possibility that even peaks that do not change position during titration (Δω∼0) may be misidentified as correlated (Figure S8B), which can be considered as a kind of statistical noise.

**Figure 6:**
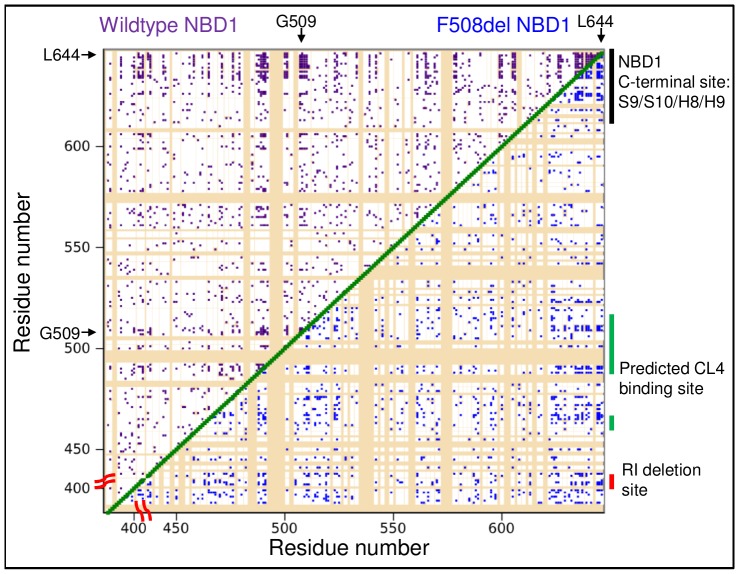
Significant inter-residue correlations due to CL4 titrations. “Contact” map of significant inter-residue correlations. Significant (p≤0.025) WT NBD1 correlations are marked in purple in the upper diagonal of the map, while those of F508del are in blue within the lower diagonal. Unassigned and unanalyzed residues are in tan. Groups of significant correlations are located in the CL4-binding, RI-deletion and NBD1 C-terminal sites.

**Figure 7:**
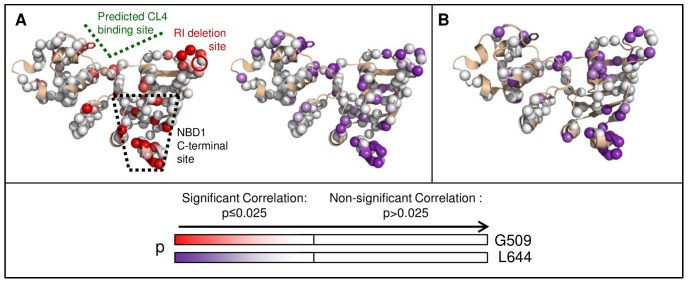
Significant inter-residue correlations due to CL4 titrations of WT and F508del NBD1. A. WT NBD1 residues that are significantly correlated with CL4-binding site residue G509 are mapped onto the NBD1 structure with a white-to-red gradient indicating increasing significance (decreasing p values). Those residues that are significantly correlated with H9 residue L644 of WT NBD1 are mapped onto the structure with an analogous white-to-purple gradient. B. Significant inter-residue correlations for the NBD1 C-terminal site residue L644 due to CL4 titrations of F508del NBD1 are mapped onto the NBD1 structure with a white-to-purple gradient. Non-significant correlations are in white, while unassigned and unanalyzed residues are in tan.

Residues with significant inter-residue correlations are found across NBD1, but many are concentrated in the CL4-binding and NBD1 C-terminal sites (Figure 6 upper diagonal). Titration data for residues with larger chemical shift changes, such as G509 in the CL4-binding site and L644 in the NBD1 C-terminal site, are the most sensitive to inter-residue correlations (Figures 6 and S8B), which allow inter-residue correlations to also be detected near the RI-deletion site. The RI-deletion site residues have sharp, narrow peaks that only move slightly during titration (Figure 3B). However, this change is still significant since these peaks have a lower than average uncertainty in peak position (Figure S8C). The many significant correlations with either G509 or L644 tend to be located near the RI-deletion, CL4-binding and NBD1 C-terminal sites (Figures 6, 7A, and S9). Correlations are also found for residues outside of the three defined regulatory sites that may link these regions or connect them to other regulatory elements, such as the ATP-binding site or the NBD1:NBD2 interface. Differences between the two sets of inter-residue correlations may be due to differences in Δω and peak position uncertainty for G509 and L644 (Figure S8C) and the statistical noise described above. These uncertainties are the most likely reasons a quantitative clustering algorithm could not be used to analyze the correlation data. Using the modified CHESCA statistical analysis, however, a third, biologically relevant site in the allosterically-coupled network was detected.

A similar pattern of inter-residue correlations emerges for F508del titration data, which demonstrates that this three-site network is a feature of NBD1 (Figures 6 lower diagonal and 7B). The residues that are significantly correlated with L644 during CL4 titration of both WT and F508del NBD1 are mapped onto the NBD1 crystal structure (Figure 7AB). While there are fewer significant F508del NBD1 correlations than for WT NBD1, a greater number of F508del NBD1 correlations are observed for parts of H5 (residues 565–572) that are adjacent to the CL4-binding site. Like WT NBD1, however, significant correlations are found near the CL4-binding site, the RI-deletion site and the NBD1 C-terminal site, highlighting the allosteric linkage between these three regions. Also importantly, we have demonstrated a method of extracting information from even relatively small chemical shift perturbations, which is of great use in a technically challenging system like CFTR.

## Discussion

A protein is composed of an interconverting ensemble of individual conformations, whose relative populations depend on their free energies [36]–[38] and can be perturbed by mutation or ligand binding. If members of the ensemble have local structural or energetic differences in regions of the protein remote from a mutation or binding site, preferential population of these members of the ensemble upon perturbation reveals allostery [36]–[38]. Therefore, an allosteric network involves coupling between disparate regions but does not necessarily require a structurally continuous pathway between these regions.

Members of the ABC transporter family are allosterically regulated by nucleotide binding at the NBDs, leading to NBD subdomain reorientations [53]–[55] and substrate binding that alters interactions with other ligands [56]–[58]. This allostery implies coupling of the NBD nucleotide-binding state to the transmembrane transporter mechanism, via interactions of the coupling helices of the membrane spanning domains to the NBDs. CFTR, the only channel in the ABC transporter family, is also allosterically regulated as a number of lines of evidence demonstrate. The importance of interactions between the NBDs and the CLs that couple them to the channel pore are reflected in the number of NBD and CL interface mutations which alter channel gating and maturation [2], [6], [7], [12], [16], [30], [59]–[63]. In single channel studies, binding of different nucleotides between the NBDs changes the gating behavior and the open channel probability [30]–[32]. The effects of allosteric coupling have been observed between the NBDs of CFTR and the rest of the channel, even in the absence of ligand or binding partner. A low basal level of channel opening occurs even after ATP is washed out or scavenged [15], [60], [64], [65] or in constructs in which NBD2 has been deleted [15], [66]. ATP-independent channel opening has been enhanced by Cys, Ser, and Pro mutations of K978 in the ICDs [15] and F1296S/N1303Q and R1358A in NBD2 [60]. Therefore, the populations of conformations required for channel function appear to be enhanced by ATP binding or mutations.

From these experiments on full-length protein, it is clear that CFTR utilizes allostery for functional regulation; however, little is known about the biophysical mechanisms of this allostery. MD simulations have demonstrated a coupling between the F508 loop in the α-subdomain and nucleotide-binding sites within NBD1 that is modulated by the CFTR-unique RI [16]. Other MD simulations of ABC transporters noted a coupling between their α-subdomains and nucleotide-binding sites [67]–[69]. Binding of ADP to apo MJ1267 NBD1 perturbs chemical shifts in the H-loop and the Walker A and B motifs of the ADP binding site, as well as in the LivG insert, which is structurally equivalent to CL binding sites of NBDs of other ABC transporter family proteins, such as the CL4-binding site in CFTR [33]. Binding enhances flexibility of the Walker A motif and the LivG insert on the ps-ns timescale, but R_ex_ values are decreased in the ADP-binding site and in the D-loop on the µs-ms timescale. These sites are well-conserved within NBDs in exporters of the ABC transporter family and show high tendency towards sequence co-evolution [13]. ATP is a major factor in the stability and solubility of CFTR NBD1 [14], however. Regulation of CFTR by its unique phospho-regulatory regions, including the RI of NBD1 and the R region C-terminal to NBD1, suggests additional allosteric coupling. Details of potential allosteric mechanisms involving these phospho-regulatory elements have not been described. Using mutations and CL4 peptide binding, in the presence of 5 mM ATP, we have explored the CFTR-specific allosteric network within NBD1 revealing coupling between the CL4-binding site, the RI-deletion site and the C-terminal site leading into the R region.

While binding assays such as ITC and CD spectroscopy have been used to demonstrate allostery in vitro [70], the low solubility of the CL4 peptide prevents the binding saturation required for these techniques. We therefore used NMR spectroscopy to characterize allostery [37]. The chemical shifts and lineshapes of NMR peaks in ^15^N-^1^H correlation experiments are sensitive, per-residue probes of changes to the conformational ensemble, even at sub-saturating ligand concentrations. Since CL4:NBD1 binding is on the fast chemical exchange timescale, the peak positions of perturbed residues shift in two dimensions during titration. However, these changes are relatively small, necessitating a more sensitive method than simple chemical shift differences to rigorously distinguish between allosterically-linked conformational changes and experimental noise. The CHESCA [51], [71] methods and projection analysis methods, such as the Co-linear Chemical Shift Perturbation (CCSP) [72] and CHESPA [71], [73], have been used to address this problem. However, both techniques reduce two dimensional titration data to one dimension, decreasing the amount of information available for analysis. Using the Fischer test [52], we were able to analyze the titration in two dimensions, increasing the detection sensitivity for correlated events in different regions of NBD1.

We found evidence for an allosteric network revealed by both the Q637R mutation and CL4 peptide binding to NBD1 that connects the CL4-binding site predicted in CFTR homology models to two regulatory sites in NBD1, near to the RI and to where the RE/R regions are located in the full-length channel. Chemical shift changes upon CL4 binding provide evidence for this allosteric network observed with minor variations in five variants of isolated NBD1–WT, F508del, F494N, V510D, and Q637R, in a CL4-NBD1 fusion protein with slightly different CL4 boundaries and in different buffer conditions. The three sites, the CL4-binding site, the RI-deletion site, and the NBD1 C-terminal site containing strands S3/S9/S10 and helices H8 and H9, are coupled via the observed allosteric network. H8 and H9 move towards random coil chemical shifts, consistent with a shift from alpha-helical to more disordered conformations. It is not possible to obtain unambiguous structural information about the conformational changes associated with binding from ^15^N-^1^H HSQC data, with the exception of helical content changes. Of note is that the allosteric network likely does not require a continuous structural pathway since mutations cannot completely disrupt the network. However, other residues outside of these defined regions are also coupled, potentially representing linkages between the regions or to other regulatory sites, such as the ATP-binding site.

The three sites in the allosteric network have previously been implicated in CFTR maturation and gating regulation. The critical role of CL4:NBD1 binding is demonstrated by perturbations of these processes by mutations on both sides of the CL4:NBD1 interface. The sidechain of F508, one of the residues in this binding site, is important for CL4:NBD1 interactions and, ultimately, for channel maturation [74]. F508del, in addition to disrupting CL4:NBD1 interactions, also destabilizes the NBD1 domain [14], [15]. The mutations that can partially suppress the folding defects of F508del NBD1 are scattered across NBD1– V510D near CL4 in the channel, I539T directly opposite the NBD1:NBD2 interface, and Q637R near the start of the R region [12], hinting at the complex allosteric nature of the NBD1 folding landscape. As with protein folding, mutations such as S492F and F508del in NBD1 and G1069R in the CL4 coupling helix perturb the gating of the channel [6], [7], [75].

The RI, a phospho-regulatory element unique to CFTR, was deleted from the NBD1 construct used in this work for improved solubility [24]. The deletion site is perturbed by both CL4 binding and the Q637R mutation between C-terminal helices H8 and H9. The heterogeneous lineshapes observed in WT and Q637R NBD1 spectra indicate that there is exchange between conformations at different timescales. The Q637R mutation has far-reaching effects on the dynamics within the ensemble, altering dynamics in several distal locations, including in the RI-deletion site and in the CL4-binding site. The RI appears to affect both the ability of the CL4 to bind NBD1 and the effects of the F508del mutation. It was initially surprising that the CL4 peptide could bind to both WT and F508del NBD1 domains lacking the RI. In full-length channels, which include the RI [8], [76], CL4 can be crosslinked to the NBD1 domain in WT CFTR, but not in F508del CFTR. The deletion of the RI from F508del CFTR partially suppresses the F508del folding defects enabling crosslinking of CL4 to F508del NBD1 [16], implying that F508del and RI can affect each other with important consequences to the binding of CL4, an interaction that may occur through the allosteric network revealed in our study. Our data mimics the RI-deleted full-length CFTR binding data and hints that F508del NBD1 binding to CL4 will be impaired only in RI-containing constructs.

The NBD1 C-terminal site is located near the NBD1:NBD2 interface, comprising strands S3/S9/S10 and helices H8/H9. The final helix, H9, may be considered part of the RE/R region. CL4 binding causes H9 peaks to move towards random coil chemical shifts, consistent with a shift from helical conformations to a more disordered ensemble. Strands S9 and S10 are uniquely conserved for CFTR within the ABC transporter family and S3/S9/S10 is the site of CFTR modulator CFFT-001 binding, also resulting in shifts of H8/H9 peaks towards random coil [35]. CFFT-001 is a dual corrector/potentiator (i.e. it can “correct” the folding defects and “potentiate” channel gating), which suggests that this allosteric network may be involved in both folding and gating function. F508del suppressor modifications are found in this region as well, including the substitution of mouse RE into human CFTR [77] and Q637R, which in combination with F494N helps suppress folding defects in CFTR [20]. The compound binding also caused small chemical shift changes in the CL4-binding site [35], as expected for sites that are allosterically linked, demonstrating the reciprocal nature of the linkage and supporting our observation that a mutation between H8 and H9, Q637R, changes the dynamics of NBD1 residues at the CL4-binding site, as well as the RI-deletion site.

It has been suggested that helical RI and RE/R region elements bound to the heterodimer interface sterically block dimerization, with phosphorylation leading to destabilization of helices and release of this block [4], [78]–[80]. Normal assembly of CFTR and an active channel require both NBD dimerization and formation of NBD:ICD contacts [11]–[13]. The coupling we observe in NBD1 between RI, the C-terminal site linking to the RE/R region and the interface with the ICD is supportive of cooperative or synergistic effects of NBD dimerization and NBD:ICD binding. Together, this points to both direct effects of regulatory phosphorylation and indirect allosteric effects in assembly of the domain-domain interfaces required for CFTR maturation and gating.

By taking advantage of the sensitivity of NMR spectroscopy and statistical correlation analysis, the technical challenges of working with weak binding of poorly soluble CFTR-derived reagents were minimized. The methods used here for distinguishing small but significant peak shifts from random error should be valuable for other difficult experimental systems, especially if binding saturation cannot be attained. Most interestingly, this approach demonstrates the existence of an allosteric network within NBD1, coupling diverse regulatory and binding sites, suggesting how these distinct inputs may be synthesized in CFTR processing and function.

## Supporting Information

Figure S1 **CL4 titrations cause similar chemical shift changes for NBD1 in two different buffers.** Chemical shift changes between apo and 500 µM CL4-bound WT NBD1 in Tris at pH 7.5 (solid black line) and sodium phosphate at pH 7.0 (dashed green line) buffer. The CL4-binding site, the RI residues deleted from the construct and the NBD1 C-terminal site are indicated with thick bars above the chart that are colored green, red, and black, respectively.
(TIF)

Figure S2 **The effects of Q637R on NBD1 chemical shifts.** Chemical shift changes in F494N NBD1 due to H8/H9 mutation Q637R limited to neighboring residues in S3/S9/S10 and H8/H9. The F494N mutation was used to improve solubility. The CL4-binding site, the RI residues deleted from the construct and the NBD1 C-terminal site are indicated with thick bars above the chart that are colored green, red, and black, respectively.
(TIF)

Figure S3 **The average chemical shift changes for CL4 peptide:WT NBD1 titration and ICL4-NBD1 fusion.** The average chemical shift change, <Δω>, increases linearly for the CL4 peptide:NBD1 titration (blue diamonds, linear fit shown as black line). The average is over residues in the CL4-binding site and the NBD1 C-terminal site. The uncertainty for <Δω> was estimated using the average uncertainty in ω_15N_ and ω_1H_ for apo 40 µM NBD1. The trendline ± uncertainty levels are indicated as dashed black lines. CL4-NBD1 fusion protein contains CL4(residues 1056–1076) connected to WT NBD1 by a (SGGG)×5 tether. The CL4 peptide used in ligand titrations (main text) contains residues 1057–1075. Tethering CL4 to NBD1 should increase the local concentration of the ligand and, hence, their binding affinity compared to 1∶1 CL4 peptide:NBD1. The <Δω> value for 1∶1 CL4 peptide:NBD1 is within the uncertainty boundaries (green triangle). The <Δω> value for CL4-NBD1 (red square) is approximately twice as great as the 1∶1 CL4 peptide:NBD1 assay, comparable to binding at 6∶1 to 8∶1 for isolated reagents. The uncertainties in <Δω> for CL4-NBD1 and 1∶1 CL4 peptide:NBD1 were estimated using the average uncertainty in ω_15N_ and ω_1H_ for 65 µM apo NBD1, 65 µM CL4 peptide: 65 µM NBD1, and 65 µM CL4-NBD1 spectra. The chemical shift changes due to CL4 binding were averaged over residues in the CL4-binding site (residues 489–492, 500, 501, 503, 507–511) and the NBD1 C-terminal site (residues 450, 453–456, 622, 623, 625–637, 639–641, 644–646) for CL4-NBD1 and the isolated NBD1 constructs. The listed residues had resolved and assigned peaks in all of the spectra analyzed here.
(TIF)

Figure S4 **Similar patterns of chemical shift changes observed for CL4 binding in WT and mutant NBD1.** Overlay of apo (black) and 12.5∶1 CL4:NBD1 (red) spectra for A. F508del NBD1, B. F494N NBD1, C. V510D NBD1, and D. Q637R NBD1 mutants.
(TIF)

Figure S5 **CL peptide binding controls.** A. CL4 peptide binding to WT NBD1 leads to small but significant chemical shift changes. B. CL1 and C. CL3 peptides have smaller observed binding to WT NBD1 based on even smaller chemical shift changes. Thick bars above charts A, B, and C indicate the CL4-binding site (green), the RI residues deleted from the construct (red) and the NBD1 C-terminal site (black). D. CL4 peptide has no observed binding to ^15^N-labeled SUMO. The SUMO spectrum has sharper peaks and a lower average chemical shift change uncertainty than NBD1 due to the protein’s smaller size and lack of line broadening.
(TIF)

Figure S6 **CL peptide binding controls.** Overlay of apo and CL peptide-bound spectra. A. apo WT NBD1(black), 12.5∶1 CL1:NBD1(purple), and 12.5∶1 CL3:NBD1(green) spectra. B. apo(black) and 12.5∶1 CL4:SUMO (red) spectra.
(TIF)

Figure S7 **L644 and F508 chemical shift titrations.** A. Correlated changes in chemical shifts for resonances of residues L644 and F508 (near the CL4-binding site) during CL4 titration, showing a selected region of HSQC spectra. B. Correlation of L644 and F508 chemical shifts in ^15^N dimension during titration. C. Correlation of L644 and F508 chemical shifts in ^1^H dimension during titration. The uncertainty of L644 ω_1H_ was found experimentally to be small compared to those of F508 ω_1H_, corresponding to error bars in the chart that are approximately the same size as the data marker.
(TIF)

Figure S8 **Test of Fischer test and CHESCA method sensitivity to small perturbations due to ligand binding.** A. Simulation setup: Linear titration curves with five points were generated for ω_1H_ and ω_15N_ coordinates. Each coordinate was simulated with a normally distributed uncertainty σ. The Fischer test and CHESCA methods differ in how the 2D coordinates are handled. In the Fischer test method, ^1^H and ^15^N titration coordinates are analyzed separately for correlations and in the CHESCA method, the weighted sum of the coordinates is analyzed. To test each method, one thousand simulations were run for each chemical shift change Δω = kσ and the percentage of simulations that found a significant correlation (p_1tail_≤0.025) were calculated. B. The Fischer test method (red) is more sensitive to correlations than the CHESCA method (black) at Δω/σ≤8 (Δω/σ = 6 is marked with a green dashed line), but has a slightly larger “background noise”. C. Most of the experimental (Δω/σ)_obs_ values for the chemical shift change between apo and 12.5∶1 CL4:WT NBD1 titration spectra are below the (Δω/σ)_obs_ = 6 threshold (marked with a green dashed line in both B and C), demonstrating the necessity of utilizing the Fischer test statistical correlation method. Thick bars above chart C indicate the CL4-binding site(green), the RI residues deleted from the construct (red) and the NBD1 C-terminal site (black).
(TIF)

Figure S9 **Significant inter-residue correlations due to CL4 titrations of WT and F508del NBD1.** Residues with significant WT NBD1 inter-residue correlations with L644 in the NBD1 C-terminal site (purple) or G509 in the CL4-binding site(red). For clarity, the (1−p) values are shown. Since the significant correlations are those with p≤0.025, the (1−p) values increase in significance from 0.975 to 1.0. The significance criterion as (1−p) = 0.975 is marked with a dotted line and below this level the chart is shaded in light grey to indicate the lack of significance of these correlations.
(TIF)

File S1 Statistical Methods for detecting inter-residue correlations in titration data.
(DOC)
