# Supplementary figures and images for: Allosteric Coupling between the Intracellular Coupling Helix 4 and Regulatory Sites of the First Nucleotide-binding Domain of CFTR
Source: PLoS One. 2013 Sep 18;8(9):e74347. doi: 10.1371/journal.pone.0074347 (PMC3776845; doi:10.1371/journal.pone.0074347)

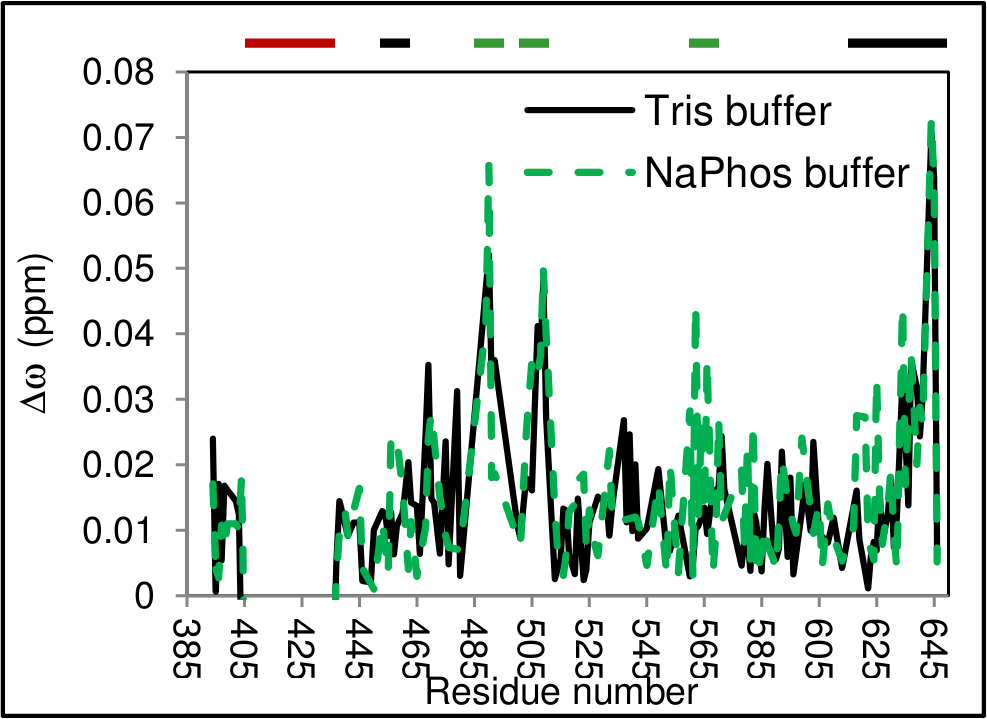

Supplement: Figure S1 — CL4 titrations cause similar chemical shift changes for NBD1 in two different buffers. Chemical shift changes between apo and 500 µM CL4-bound WT NBD1 in Tris at pH 7.5 (solid black line) and sodium phosphate at pH 7.0 (dashed green line) buffer. The CL4-binding site, the RI residues deleted from the construct and the NBD1 C-terminal site are indicated with thick bars above the chart that are colored green, red, and black, respectively. (TIF) [file pone.0074347.s001.tif]

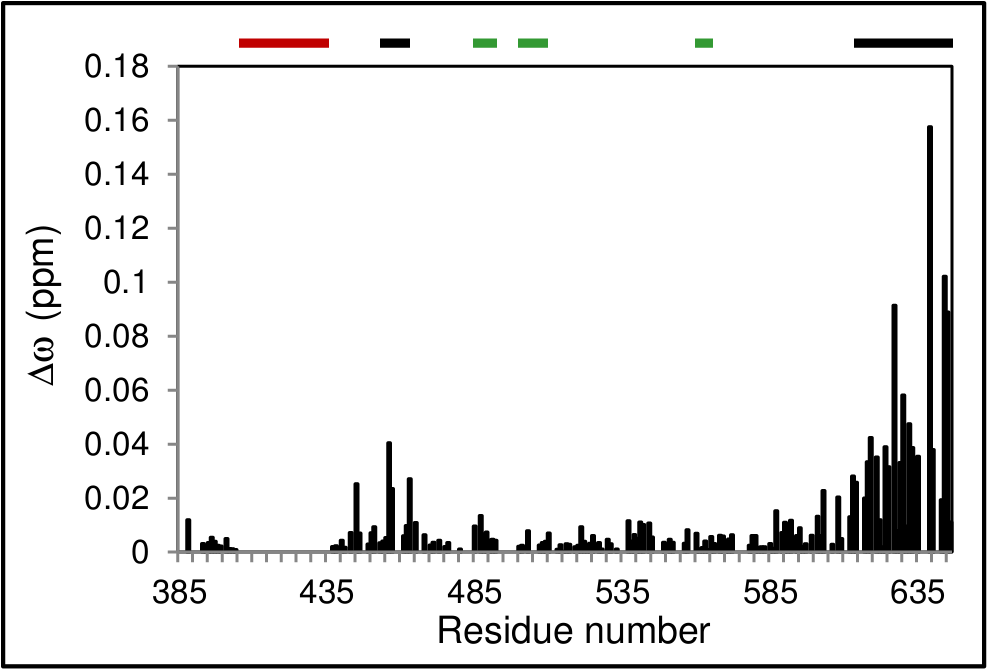

Supplement: Figure S2 — The effects of Q637R on NBD1 chemical shifts. Chemical shift changes in F494N NBD1 due to H8/H9 mutation Q637R limited to neighboring residues in S3/S9/S10 and H8/H9. The F494N mutation was used to improve solubility. The CL4-binding site, the RI residues deleted from the construct and the NBD1 C-terminal site are indicated with thick bars above the chart that are colored green, red, and black, respectively. (TIF) [file pone.0074347.s002.tif]

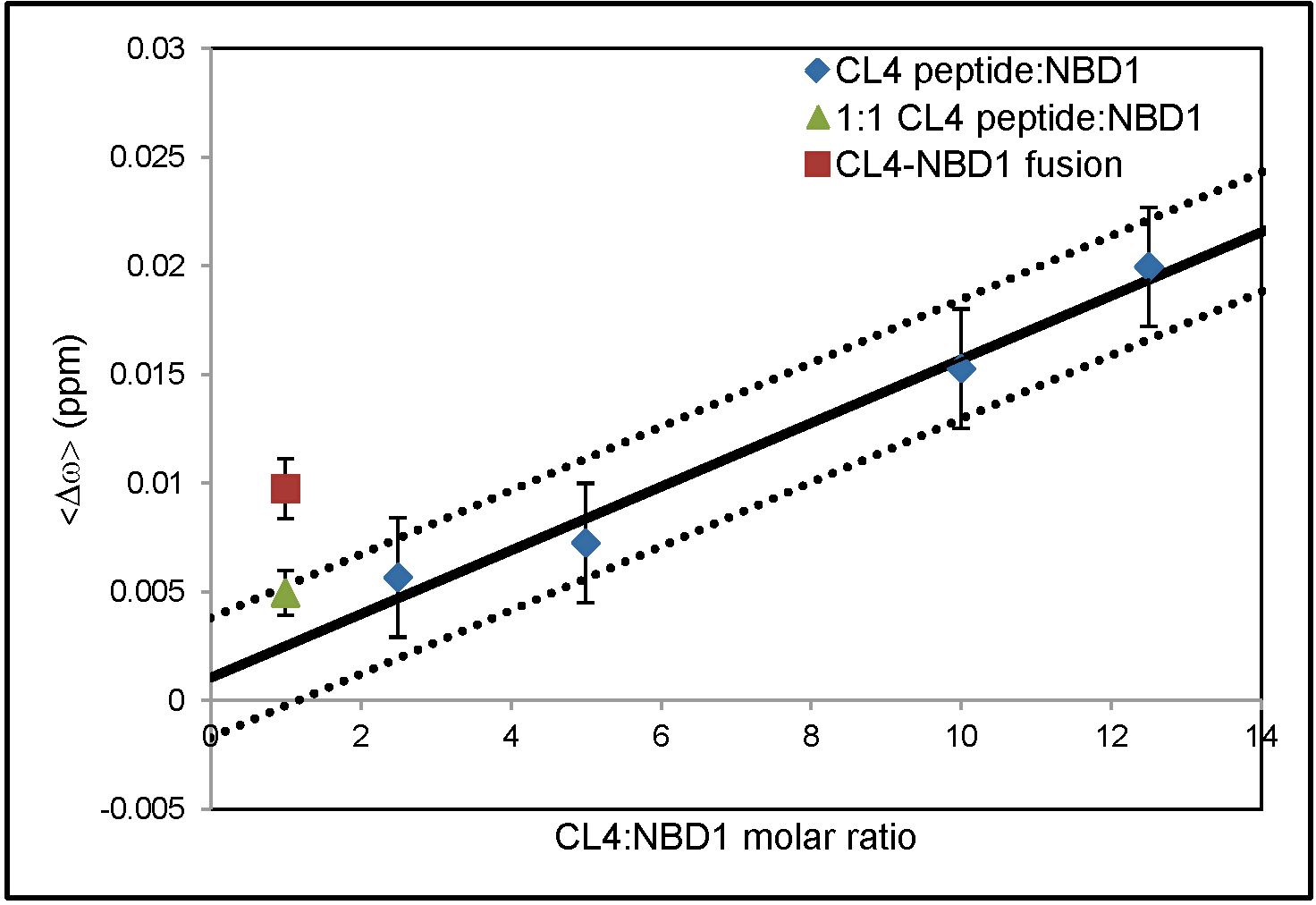

Supplement: Figure S3 — The average chemical shift changes for CL4 peptide:WT NBD1 titration and ICL4-NBD1 fusion. The average chemical shift change, <Δω>, increases linearly for the CL4 peptide:NBD1 titration (blue diamonds, linear fit shown as black line). The average is over residues in the CL4-binding site and the NBD1 C-terminal site. The uncertainty for <Δω> was estimated using the average uncertainty in ω15N and ω1H for apo 40 µM NBD1. The trendline ± uncertainty levels are indicated as dashed black lines. CL4-NBD1 fusion protein contains CL4(residues 1056–1076) connected to WT NBD1 by a (SGGG)×5 tether. The CL4 peptide used in ligand titrations (main text) contains residues 1057–1075. Tethering CL4 to NBD1 should increase the local concentration of the ligand and, hence, their binding affinity compared to 1∶1 CL4 peptide:NBD1. The <Δω> value for 1∶1 CL4 peptide:NBD1 is within the uncertainty boundaries (green triangle). The <Δω> value for CL4-NBD1 (red square) is approximately twice as great as the 1∶1 CL4 peptide:NBD1 assay, comparable to binding at 6∶1 to 8∶1 for isolated reagents. The uncertainties in <Δω> for CL4-NBD1 and 1∶1 CL4 peptide:NBD1 were estimated using the average uncertainty in ω15N and ω1H for 65 µM apo NBD1, 65 µM CL4 peptide: 65 µM NBD1, and 65 µM CL4-NBD1 spectra. The chemical shift changes due to CL4 binding were averaged over residues in the CL4-binding site (residues 489–492, 500, 501, 503, 507–511) and the NBD1 C-terminal site (residues 450, 453–456, 622, 623, 625–637, 639–641, 644–646) for CL4-NBD1 and the isolated NBD1 constructs. The listed residues had resolved and assigned peaks in all of the spectra analyzed here. (TIF) [file pone.0074347.s003.tif]

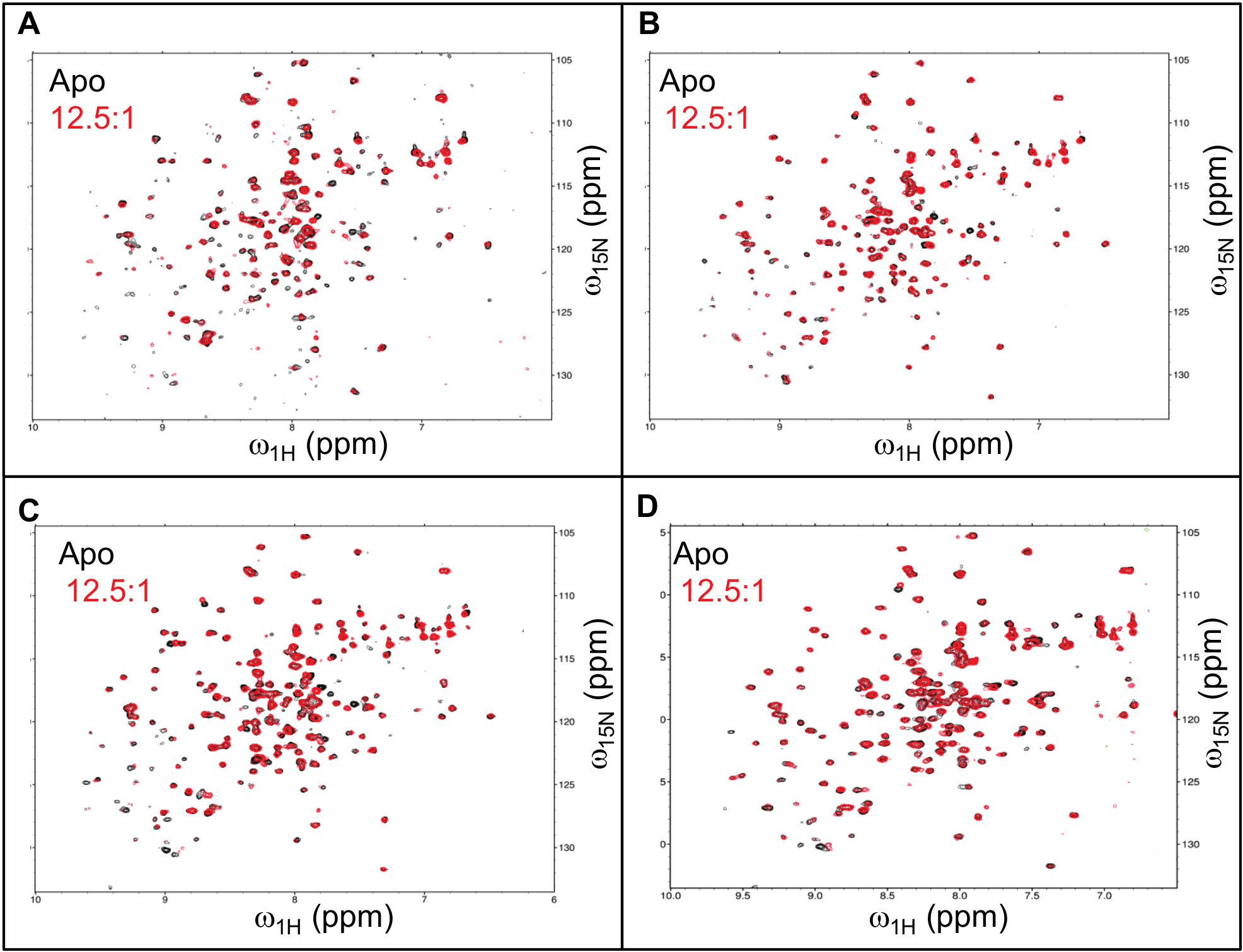

Supplement: Figure S4 — Similar patterns of chemical shift changes observed for CL4 binding in WT and mutant NBD1. Overlay of apo (black) and 12.5∶1 CL4:NBD1 (red) spectra for A. F508del NBD1, B. F494N NBD1, C. V510D NBD1, and D. Q637R NBD1 mutants. (TIF) [file pone.0074347.s004.tif]

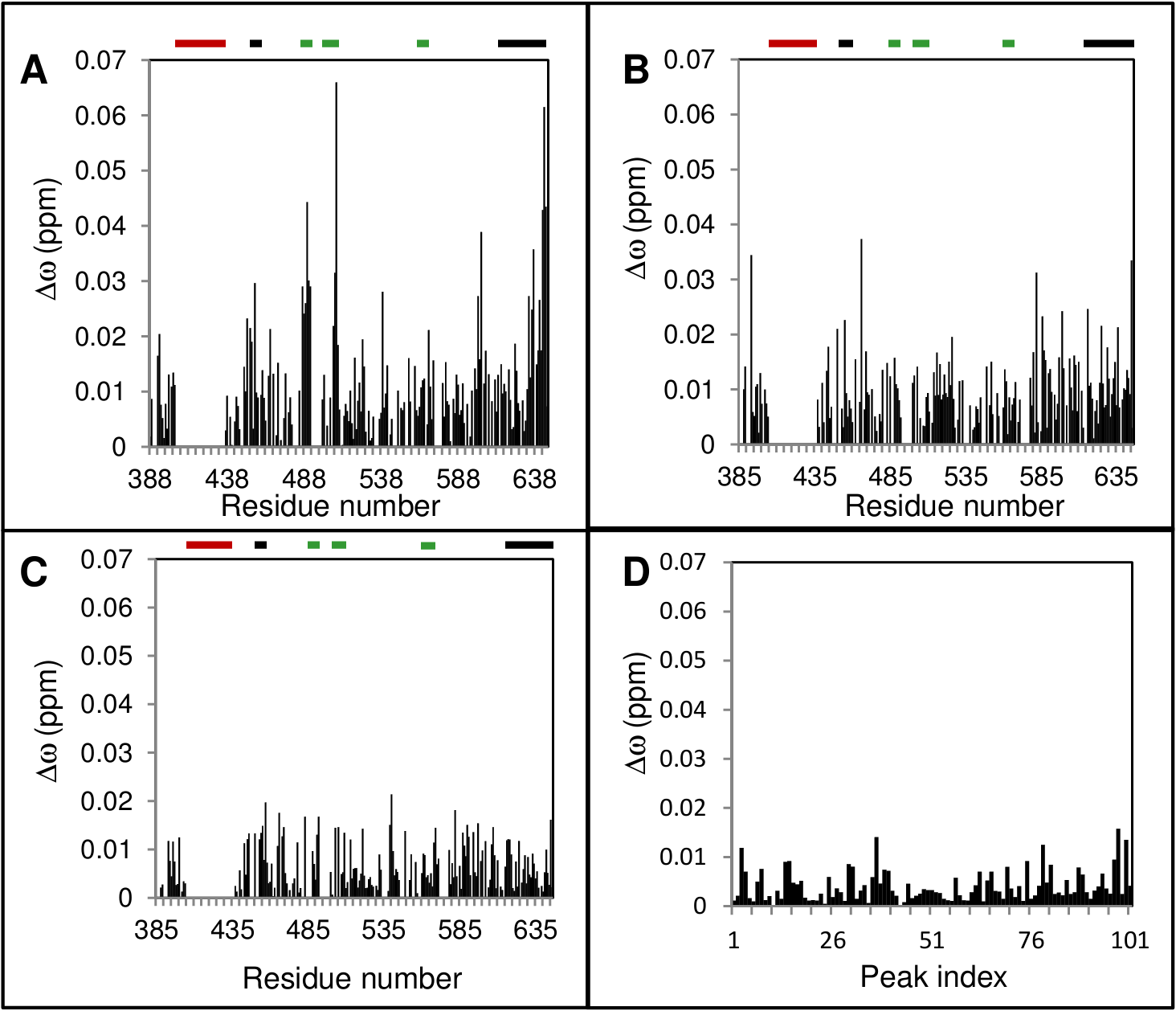

Supplement: Figure S5 — CL peptide binding controls. A. CL4 peptide binding to WT NBD1 leads to small but significant chemical shift changes. B. CL1 and C. CL3 peptides have smaller observed binding to WT NBD1 based on even smaller chemical shift changes. Thick bars above charts A, B, and C indicate the CL4-binding site (green), the RI residues deleted from the construct (red) and the NBD1 C-terminal site (black). D. CL4 peptide has no observed binding to 15N-labeled SUMO. The SUMO spectrum has sharper peaks and a lower average chemical shift change uncertainty than NBD1 due to the protein’s smaller size and lack of line broadening. (TIF) [file pone.0074347.s005.tif]

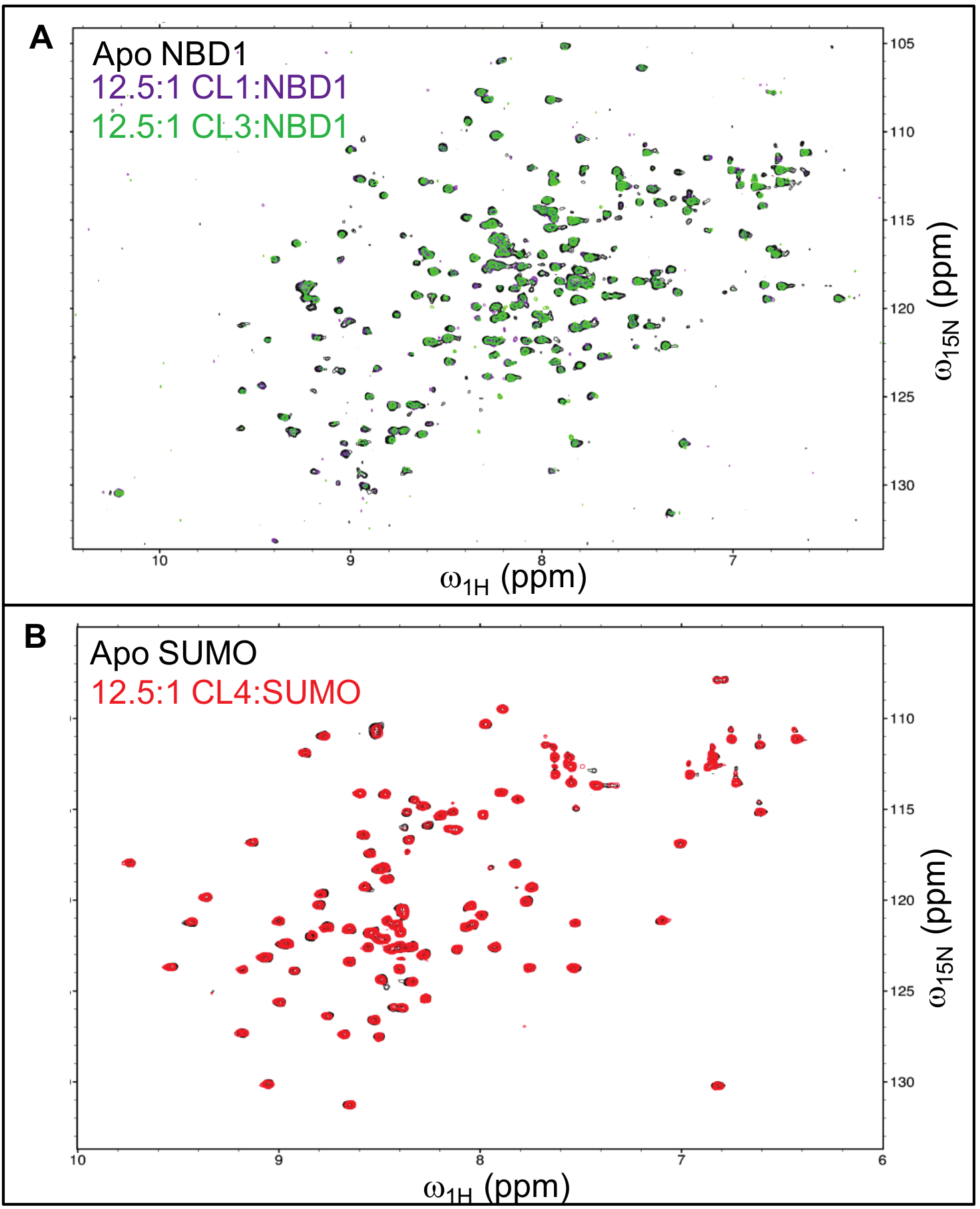

Supplement: Figure S6 — CL peptide binding controls. Overlay of apo and CL peptide-bound spectra. A. apo WT NBD1(black), 12.5∶1 CL1:NBD1(purple), and 12.5∶1 CL3:NBD1(green) spectra. B. apo(black) and 12.5∶1 CL4:SUMO (red) spectra. (TIF) [file pone.0074347.s006.tif]

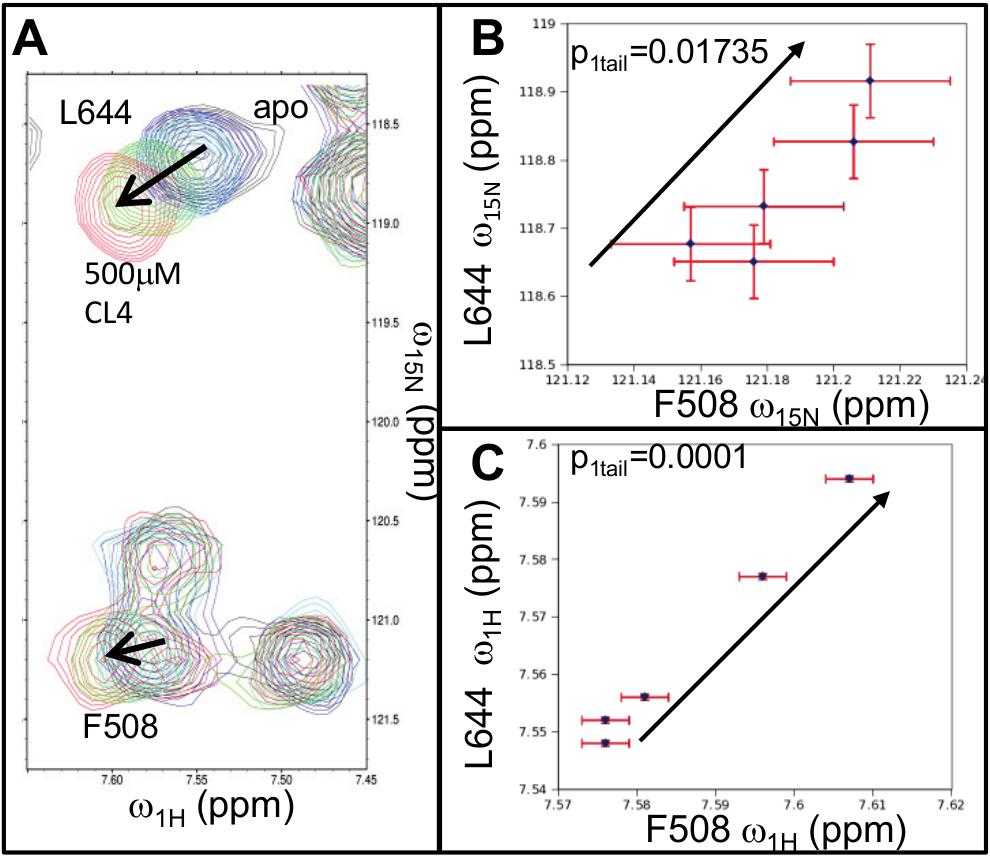

Supplement: Figure S7 — L644 and F508 chemical shift titrations. A. Correlated changes in chemical shifts for resonances of residues L644 and F508 (near the CL4-binding site) during CL4 titration, showing a selected region of HSQC spectra. B. Correlation of L644 and F508 chemical shifts in 15N dimension during titration. C. Correlation of L644 and F508 chemical shifts in 1H dimension during titration. The uncertainty of L644 ω1H was found experimentally to be small compared to those of F508 ω1H, corresponding to error bars in the chart that are approximately the same size as the data marker. (TIF) [file pone.0074347.s007.tif]

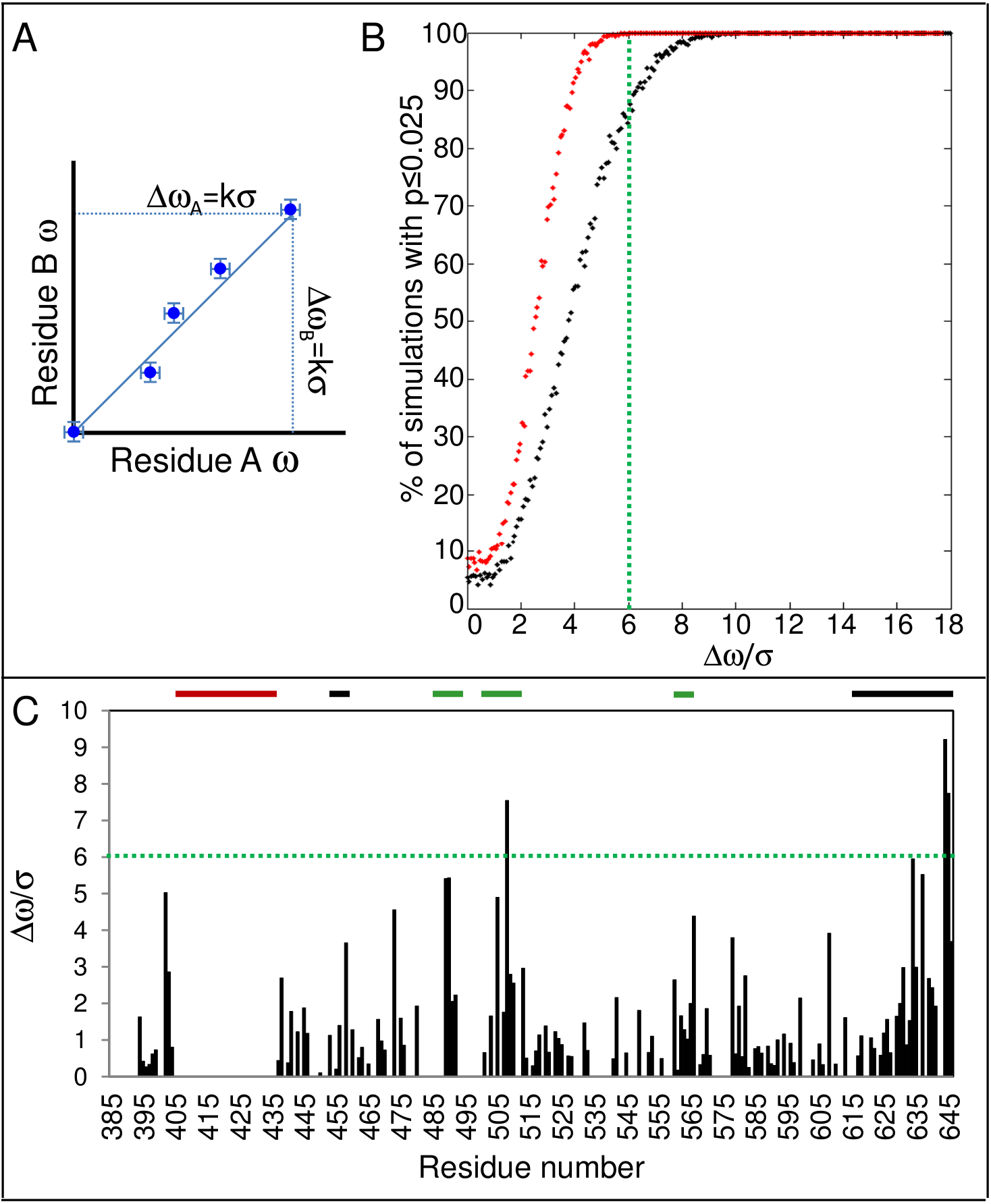

Supplement: Figure S8 — Test of Fischer test and CHESCA method sensitivity to small perturbations due to ligand binding. A. Simulation setup: Linear titration curves with five points were generated for ω1H and ω15N coordinates. Each coordinate was simulated with a normally distributed uncertainty σ. The Fischer test and CHESCA methods differ in how the 2D coordinates are handled. In the Fischer test method, 1H and 15N titration coordinates are analyzed separately for correlations and in the CHESCA method, the weighted sum of the coordinates is analyzed. To test each method, one thousand simulations were run for each chemical shift change Δω = kσ and the percentage of simulations that found a significant correlation (p1tail≤0.025) were calculated. B. The Fischer test method (red) is more sensitive to correlations than the CHESCA method (black) at Δω/σ≤8 (Δω/σ = 6 is marked with a green dashed line), but has a slightly larger “background noise”. C. Most of the experimental (Δω/σ)obs values for the chemical shift change between apo and 12.5∶1 CL4:WT NBD1 titration spectra are below the (Δω/σ)obs = 6 threshold (marked with a green dashed line in both B and C), demonstrating the necessity of utilizing the Fischer test statistical correlation method. Thick bars above chart C indicate the CL4-binding site(green), the RI residues deleted from the construct (red) and the NBD1 C-terminal site (black). (TIF) [file pone.0074347.s008.tif]

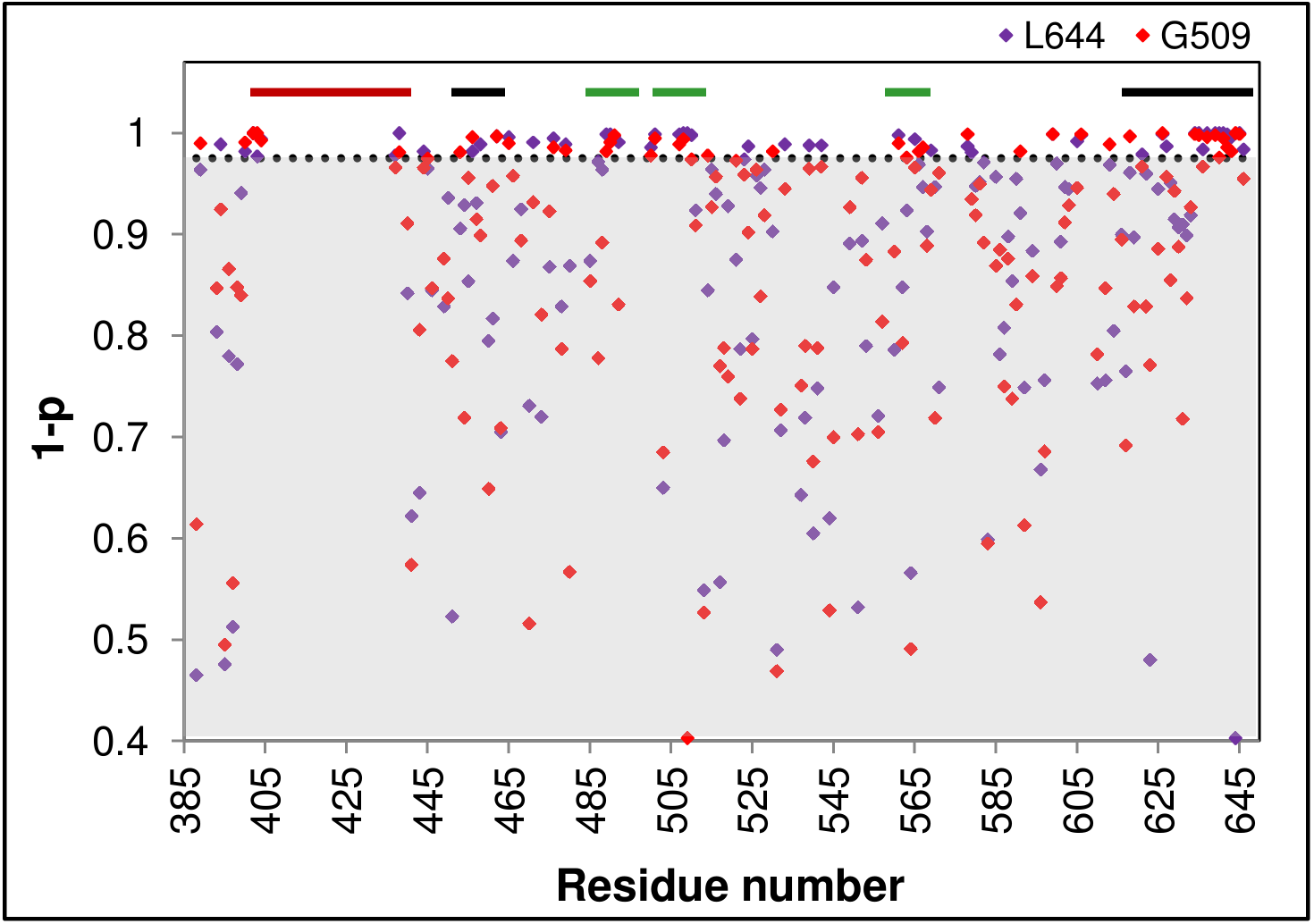

Supplement: Figure S9 — Significant inter-residue correlations due to CL4 titrations of WT and F508del NBD1. Residues with significant WT NBD1 inter-residue correlations with L644 in the NBD1 C-terminal site (purple) or G509 in the CL4-binding site(red). For clarity, the (1−p) values are shown. Since the significant correlations are those with p≤0.025, the (1−p) values increase in significance from 0.975 to 1.0. The significance criterion as (1−p) = 0.975 is marked with a dotted line and below this level the chart is shaded in light grey to indicate the lack of significance of these correlations. (TIF) [file pone.0074347.s009.tif]
