# Supplementary material for: Allosteric Coupling between the Intracellular Coupling Helix 4 and Regulatory Sites of the First Nucleotide-binding Domain of CFTR
Source: PLoS One. 2013 Sep 18;8(9):e74347. doi: 10.1371/journal.pone.0074347 (PMC3776845; doi:10.1371/journal.pone.0074347)
Supplement: File S1 — Statistical Methods for detecting inter-residue correlations in titration data. (DOC) [file pone.0074347.s010.doc]

**Supplementary materials**

*Statistical Methods for detecting inter-residue correlations in titration data*

Peak position, ω, can be affected by both proximal ligand binding and long-distance conformational changes that alter the relative populations within an ensemble. For a residue that is affected by ligand binding on the fast chemical exchange timescale, its peak position can shift in two dimensions during titration. When ligand binding causes relatively large and unambiguous chemical shift changes for perturbed residues, the simplest method of analyzing titration data is generally to evaluate the distance (Δω_obs_) that the protein’s peaks move upon addition of ligand. Only two NMR spectra are required (apo and bound), though additional spectra are of help with peak identification. However, as the perturbation of the peaks becomes smaller, statistical analysis methods become increasingly useful. Residues perturbed by binding should have correlated chemical shift changes and the inclusion of spectra from multiple points in the titration into the statistical analysis to reduce the effects of experimental noise.

If there is allosteric coupling between disparate regions of the protein, then chemical shift changes during titration should be correlated between affected residues. This idea was powerfully applied previously using the CHESCA (CHEmical Shift Correlation Analysis) method. With this approach, each residue’s titration path in two dimensions is reduced to a single dimension by taking the weighted sum of the ^1^H and ^15^N coordinates at each titration point.

ω = ω_1H_ + 0.154*ω_15N_. Eq. S1

The titration paths of residues A and B are considered to be correlated if their covariance is significantly different from that expected due to random uncertainty. The significance of a correlation is described by its two-tailed p-value, p_2tail_, for normally distributed uncertainty. In this paper, we present a modification to the CHESCA method, incorporating a Fischer combined probability test, with improved sensitivity for detecting correlations between titrating residues, especially for those with very small chemical shift changes.

In the Fischer test method, the chemical shift changes in the ^15^N and ^1^H dimensions (Figure S7) are treated as two independent tests of correlation. If multiple independent tests of the same correlation are available (i.e. multiple probes of the same physical process or repeated data sets), then a Fischer test can be used to combine the p_2tail_ results of each correlation test. Before the Fischer test can be used, each p_2tail_ must be converted into a p_1tail_ value. Since no distinction is made between correlated and anti-correlated titration paths, p_1tail_=p_2tail_/2. The test statistic, F, combines the p_1tail_ values from k independent tests.

$F=-2*\sum_{i=1}^{k} \ln(p_{1tail,i})$ Eq. S2

The Fischer test uses the cumulative chi-squared distribution (described by the incomplete Gamma function with k degrees of freedom for F/2) and yields a one-tailed p-value for correlation between the pair of residues, p_F_. The possibility that titrations of two residues are uncorrelated may be rejected if p_F_≤p_crit_, the significance criterion. A value of p_crit_=0.025 was used throughout the statistical analysis of the CL4 titration data in the main text. The experimental data discussed in the main text was analyzed using the Fischer test method, which was implemented in Matlab (files available by request).

*Simulation testing the CHESCA and Fisher test statistical correlation sensitivity*

The Fischer test and the CHESCA statistical methods differ in how peak coordinates are handled. For titration assays performed using ^15^N-^1^H HSQC spectra, the CHESCA method takes a weighted sum of the ^1^H and ^15^N chemical shifts for each peak and then analyzes for correlations, resulting in a two-tail p-value reporting on the significance of the correlation. The Fischer test method builds on the CHESCA method. In the Fischer test method, titrations in each dimension are analyzed separately for correlations, and then their p-values combined in combined probability Fischer test, resulting in a one-tailed p-values.

The sensitivity of each technique for correlated titrations was tested *in silico* through a range of chemical shift change magnitudes (Figure S8A). Sets of titration peak coordinates (ω_1H_,ω_15N_) were simulated for two residues, A and B. Each set contained five evenly-distributed points simulating a titration series from the apo to the final titration point. The choice of five points reflects the five titration spectra in the CL4/wildtype NBD1 data set examined in the main text. For simplicity, the ω_1H_ and ω_15N_ coordinates of each point in the titration were set to the same value, forming a line running at a 45° angle in a two-dimensional plane. Additionally, residues A and B were assumed to have equal chemical shift differences between the first and final titration points: Δω=Δω_A_=Δω_B_. Noise was added to each point in the two residue simulation using a normally-distributed uncertainty with a standard deviation of σ. In order to simulate ligand binding that causes only relatively small perturbations to any of the affected residues, Δω was varied from 0 to 25σ.

For the simulation of the CHESCA method, ω_1H_ and ω_15N_ for each titration point were added in a weighted sum (Eq. S1). A factor of 0.2 was used in ref , but in this simulation 0.154 was employed to maintain consistency with the weighting used to calculate the Δω_obs_ values of the experimental CL/NBD1 data in the main text. The simulated curves were tested for linear correlations, yielding a two-tailed p-value reflecting the significance of the correlation. In order to compare the results of the Fischer test simulation, 2-tailed p-value for the correlation was converted to a one-tailed value by dividing it in half.

The ω_1H_ and ω_15N_ coordinates were considered separately for the Fischer test simulation. For a given value of Δω, a pair of two-tailed p-values was obtained for correlations in the ^1^H and ^15^N dimensions. The pair was converted to one-tailed p-values by dividing them by two and combining them in a Fischer test, yielding a one-tailed p-value.

For both the CHESCA and Fischer test simulations, the correlation test was repeated 1000 times for a given value of Δω. The percentage of simulations with significant correlations (p≤0.025) gives an estimate of how well a residue with a given Δω can detect a small but significant titration. Summing of the ^1^H and ^15^N coordinates during the CHESCA simulations scales up both the magnitude of the chemical shift change and the uncertainty, which must be accounted for before these simulations can be compared to those of the Fischer test. Since ω_1H_ and ω_15N_ are set equal to each other, the magnitude is increased by a factor of (1+0.154) and the uncertainty increases by (2*{1+0.154^2^})^1/2^. To compare the sensitivities of the two statistical correlation methods, the Δω/σ values used in Figure S8B were scaled by (1+0.154)/(2*{1+0.154^2^})^1/2^ for the CHESCA simulations and by 1/√2 for the Fischer test. The simulations were implemented with Matlab and are available upon request.

The Fischer test has a greater baseline noise, i.e. the likelihood that a randomly moving peak will be identified as having a significant correlation with another residue’s titration path (Figure S8B). More importantly, it was found that correlation detection depends on the magnitude of the chemical shift change for both methods. The Fischer combined probability method is predicted to be more sensitive than CHESCA to correlated titrations with small Δω relative to the noise σ, i.e. those Δω that are only greater than the noise by a factor of eight or less (Δω/σ≤8).

As a comparison, the experimental (Δω/σ)_obs_ values were calculated for the chemical shift changes between the apo and 12.5:1 CL4-bound HSQC spectra of 40 μM WT NBD1 (Figure S8C). The Δω_obs_ values were calculated using Eq. 1 (main text). The σ_obs_ values were estimated by finding the standard deviation of each peak’s position, ω, in three repeated HSQC spectra of the same apo 40 μM WT NBD1 sample, then propagating the error by assuming that the uncertainties in apo peak position are roughly equivalent to those in the CL4-bound spectrum. This assumption would most likely result in an underestimation of σ_obs_ values because of the general decrease in peak intensities due to CL4 binding. Most of the (Δω/σ)_obs_ values are below (Δω/σ)_obs_ =6, demonstrating, in general, the need for statistical analysis of the titration data and more specifically and importantly, the necessity of utilizing the Fischer test statistical correlation method.

*Application of the Fischer test method to WT and F508del NBD1 CL4 titration data*

A single set of 2D HSQC spectra recorded at 600 MHz are available for the CL4:WT NBD1 titration. In this case, the Fischer test statistic for each inter-residue correlation is

$F=-2*\left[\ln(p_{1tail}^{1H})+\ln(p_{1tail}^{15N})\right]$. Eq. S3a

Two sets of titration data (collected at 600 and 800 MHz) were used for statistical analysis of the CL4: F508del NBD1 binding. Therefore, for the F508del NBD1 data, the test statistic has four terms:

$F=-2*\left[\ln(p_{1tail}^{1H,set1})+\ln(p_{1tail}^{15N,set1})+\ln(p_{1tail}^{1H,set2})+\ln(p_{1tail}^{15N,set2})\right]$. Eq. S3b

Pairs of NBD1 residues with significantly correlated CL4 titrations (p_F_≤0.025) are marked in a “contact map” (Figure 6). Amidst the scattering of inter-residue correlations that form the “background noise” of the statistical analysis, there are three major clusters of inter-residue correlations, centered in the CL4 binding site, the NBD1 C-terminal site, and the RI deletion site.

**References**

1 Selvaratnam R, Chowdhury S, VanSchouwen B, Melacini G (2011) Mapping allostery through the covariance analysis of NMR chemical shifts. Proceedings of the National Academy of Sciences of the United States of America 108: 6133-6138.

2 Fischer RA (1932) Statistical Methods for Reseach Workers.

3 Seavey BR, Farr EA, Westler WM, Markley JL (1991) A relational database for sequence-specific protein NMR data. Journal of Biomolecular NMR 1: 217-236.
